# Insight Into Disorder, Stress and Strain of Radiation Damaged Pyrochlores: A Possible Mechanism for the Appearance of Defect Fluorite

**DOI:** 10.3389/fchem.2021.706736

**Published:** 2021-11-08

**Authors:** Sarah C. Finkeldei, Shirley Chang, Mihail Ionescu, Daniel Oldfield, Joel Davis, Gregory R. Lumpkin, David Simeone, Max Avdeev, Felix Brandt, Dirk Bosbach, Martina Klinkenberg, Gordon J. Thorogood

**Affiliations:** ^1^ Department of Chemistry, University of California, Irvine, Irvine, CA, United States; ^2^ Department of Chemical and Biomolecular Engineering, University of California, Irvine, Irvine, CA, United States; ^3^ Department of Materials Science and Engineering, University of California, Irvine, Irvine, CA, United States; ^4^ Australian Nuclear Science and Technology Organisation, Lucas Heights, NSW, Australia; ^5^ CEA/DEN/DMN/SRMA/LA2M-LRC CARMEN, CEA, Université Paris-Saclay, Gif-sur-Yvette, France; ^6^ Forschungszentrum Juelich GmbH, Institute of Energy and Climate Research, IEK-6: Nuclear Waste Management and Reactor Safety, Juelich, Germany; ^7^ Department of Nuclear System Safety Engineering, Nagaoka University of Technology, Nagaoka, Japan

**Keywords:** nuclear waste form materials, radiation damage and others irradiation effects, order/disorder phase transition, pyrochlore, stress, neutron diffaction, transmission electron microscopy

## Abstract

We have examined the irradiation response of a titanate and zirconate pyrochlore—both of which are well studied in the literature individually—in an attempt to define the appearance of defect fluorite in zirconate pyrochlores. To our knowledge this study is unique in that it attempts to discover the mechanism of formation by a comparison of the different systems exposed to the same conditions and then examined *via* a range of techniques that cover a wide length scale. The conditions of approximately 1 displacement per atom *via* He^2+^ ions were used to simulate long term waste storage conditions as outlined by previous results from Ewing in a large enough sample volume to allow for neutron diffraction, as not attempted previously. The titanate sample, used as a baseline comparison since it readily becomes amorphous under these conditions behaved as expected. In contrast, the zirconate sample accumulates tensile stress in the absence of detectable strain. We propose this is analogous to the lanthanide zirconate pyrochlores examined by Simeone et al. where they reported the appearance of defect fluorite diffraction patterns due to a reduction in grain size. Radiation damage and stress results in the grains breaking into even smaller crystallites, thus creating even smaller coherent diffraction domains. An (ErNd)_2_(ZrTi)_2_O_7_ pyrochlore was synthesized to examine which mechanism might dominate, amorphization or stress/strain build up. Although strain was detected in the pristine sample *via* Synchrotron X-ray diffraction it was not of sufficient quality to perform a full analysis on.

## Introduction

Pyrochlores cover a large variation of compositions (Subramanian et al., 1983) and there have been numerous publications that endeavor to understand the tolerance pyrochlores have to radiation damage (Lian et al., 2002; Sattonnay et al., 2008; Pilania et al., 2017; Holesinger et al., 2019). The reason for this goes well beyond the application to immobilize nuclear waste (Lumpkin and Ewing, 1985; Ewing et al., 2004) and the subsequent amorphization (Lang et al., 2009). Due to properties such as colossal magnetoresistance (Subramanian et al., 1996), ion conduction (Subramanian et al., 1985; Heremans et al., 1995; Pirzada et al., 2001), and superconductivity (Yonezawa et al., 2004) pyrochlores and their variations have many applications such as thermal barrier coatings (Lehmann et al., 2003), fuel cells (Bae and Steele, 1999), and thermo-electrics (Rao et al., 1986). All of these applications and properties are related in some way to the disorder within the system being either engineered or making use of the materials resistance to it (Simeone et al., 2017). To study pyrochlores as potential nuclear waste forms, accelerated radiation damage to simulate the effects caused by either fission fragments or the interactions with neutrons (Nordlund et al., 2015) is achieved by ion irradiation of a pyrochlore’s near surface. Characterization of the material is typically performed *ex situ* since ions are generally produced by an accelerator. One of the issues with using ions is that they have a very small interaction volume when compared with the bulk of the material and so typically produce a very thin layer and thus small amount of damaged material to be examined, utilizing techniques such as Glancing Incidence X-Ray Diffraction (GIXRD) (Li et al., 2012) or Transmission Electron Microscopy (TEM) (Lian et al., 2002). More recently there have been attempts to combine other techniques such as spectroscopy with GIXRD and TEM (Lenz, 2019). In an attempt to better understand the order/disorder process up to the amorphization of the structure and how that may also be related to the stress and strain imparted, we have irradiated two well studied pyrochlores Er_2_Ti_2_O_7_ (routinely reported as transforming to amorphous material under irradiation) (Sickafus KE. et al., 2000; Li et al., 2012) and Nd_2_Zr_2_O_7_ (routinely reported as transforming to a defect fluorite under irradiation) (Lutique et al., 2003). He^2+^ ions were used to capitalize on the wealth of data already in the literature (Zhang et al., 2015; Hu et al., 2018). The reasoning behind the choice of the two pyrochlores was guided by the fact that as *R*_*A*_ */R*_*B*_ decreases, the pyrochlore structure becomes less stable (Helean et al., 2004). Helean et al. performed a comprehensive study where they examined the relationship between *R*_*B*_ and $\Delta H_{f-ox}$ for titanates at 298 K and found that it is non-linear and approximately parallels the increasing “resistance” to ion-beam-induced amorphization as *R*_*A*_ */R*_*B*_ decreases. For example, Lumpkin et al. (2009) when irradiating Nd_2_Zr_2_O_7_ with 1.0 MeV Kr ions reported a *Tc* of 135 K as compared to the *Tc* value of 804 K for Er_2_Ti_2_O_7_ as reported by Helean et al. (2004) indicating that Nd_2_Zr_2_O_7_ has a higher resistance to amorphization as compared to Er_2_Ti_2_O_7_. Combined characterization *via* neutron diffraction commonly used for bulk materials (in this study applied to approximately one quarter of a 100 micron thin sample as noted by EBSD), electron backscatter diffraction (EBSD) as used in surface examination and TEM, typically a high-resolution technique, enabled mechanistic insights into how the disorder process proceeds. Fabrication of a solid solution (ErNd)_2_(TiZr)_2_O_7_ sample was predicted to provide additional information on the dominating behavior within a solid solution. However, due to the presence of a second phase only preliminary studies were performed on the solid solution sample. There are two main advantages to using He^2+^ ions for radiation damage, the first being that the accelerating voltage of the ion can be varied to allow for damage over a range of depths thus achieving what would be considered bulk damage in a very thin section of the material. Second, the use of He^2+^ ions for irradiation is a simulant for alpha particles, a common fission fragment found in nuclear waste forms and nuclear fuel. Current literature related to radiation damage lists the amount of damage as displacements per atom (dpa). In this study we aimed for damage of approximately 1 dpa to examine the onset of disorder. Being able to vary the range of irradiation depths on a thin pellet allowed us to obtain a “bulk” amount or significant proportion of radiation-damaged material to perform high-resolution neutron diffraction as well as analysis of subsequent subsamples for EBSD and TEM. This approach mimics typical radiation damage e.g. caused by alpha particles, which occurs throughout the waste form or fuel rather than solely at the surface. The effects of damage due to alpha particles in a bulk pyrochlore has not been studied due to the above stated challenges. Typically to understand this type of damage solid solutions are used with simulants to impart disorder and then conclusions are drawn as to what types of phase changes may occur. Here, neutron diffraction data obtained from the damaged samples was analyzed to determine the difference in disorder and the behavior of the cations vs. the anions as well as to compare the degree of stress and strain in the damaged material with the undamaged material.

In reviewing the literature there are different ways that the effect of He^2+^ ions being accelerated at a ceramic can be envisaged. If we consider each interaction of the He^2+^ ions as ballistic phenomena as per Scapin (Martina Scapin, 2013) where the interaction is considered a high strain rate case they have stated that the material is in a hydrostatic state, in which the three normal components of stress are all equal to the pressure (opposite in sign). This also implies that the three normal components of strain are equal. This leads to the assumption that the pressure is proportional to the volumetric strain, and the scale factor is represented by the bulk modulus. The hydrostatic component of stress is associated to the pressure in the material, which is equal to the trace of the complete stress tensor. Ceramics are characterized by having a small plastic deformation and nearly flat fracture surfaces, which originates from a single crack propagation. Scapin (Martina Scapin, 2013) also envisioned the He^2+^ ions as a pressure wave incident on the sample the pressure can be used as failure parameter defining a failure model (i.e., accelerating voltage of the ions). The spalling defines the failure of a material in the case of high hydrostatic tensile loads, which usually characterize all the phenomena in which a high compressive wave is reflected into a tensile one by a free surface. The part of the component behind the impacted area (i.e., below the SRIM calculated depth) could be subjected to considerable values of tensile hydrostatic stresses, in accordance with the propagation of cylindrical pressure shock-waves (Martina Scapin, 2013).

Researchers at CERN have taken a similar approach in their study of beam-induced damage mechanisms and their calculation (Bertarelli, 2014), where they have made the assumption of purely hydrostatic (fluid-like) behavior of the impacted solids (each ion interaction can be imagined a cone shaped shock wave moving forward), which is typically acceptable when the achieved stresses greatly exceed the flow strength of the material and the stress tensor can be approximately reduced to its hydrostatic component only; nowadays, the deviatoric component, responsible for material strength. This internal strain energy can be partitioned into one part associated with volume change (caused by hydrostatic stress, *σ*_avg_ = (*σ*1 + *σ*2 + *σ*3)/3 and another part associated with distortion of the shape of the material element by the remaining portion of the principal stresses, corresponding to the deviatoric components of the stress tensor.

The determination of hydrostatic stress *via* diffraction has been applied to several material types such as ceramics; Bartolomé et al. (2008), Sattonnay et al. (2007), as well as irradiated polymers; Mallick et al. (2006). Also in some alloys, an analogous behavior is observed as per Rösler et al. (2007). The stainless steel X5CrNi18-10, which is austenitic (face-centred cubic) at room temperature, is only metastable. Thus, the ferritic phase is thermodynamically stable, but the transformation does not occur because the driving force is too small. Under mechanical load, for instance during forming, a martensitic transformation can take place in parts of the component. Due to this characteristic Parikin et al. (2011), Maimaitiyili et al. (2019) have also calculated the hydrostatic stress. As per Sattonnay et al. (2007) if the material is elastically isotropic, only two elastic constants, the Young modulus and the Poisson ratio, are required to describe the elastic behavior of the material in any state of stress. According to Bartolomé et al. (2008) in their study “neutron diffraction residual stress analysis of zirconia toughened alumina (ZTA) composites”, they stated that when the composite is cooled from the sintering temperature, the reinforcement contracts more than the matrix. This resulted in tensile stress in the particles and compressive stress in the matrix. Since the particles were nearly spherical in form the mean field stress is generally hydrostatic. Bartolomé et al. (2008) highlighted the work of Wang et al. (1994), Alexander et al. (1995) where they studied the internal stresses and transformations of Al_2_O_3_/Ce-TZP as function of zirconia volume fraction. In their study they used a powder diffractometer and consequently measured the average strain and hence assumed the stress to be hydrostatic.

In this study to compare the strain measurements derived from the neutron data, synchrotron data was obtained from the pristine pellets, the strain values were determined and subsequently compared with the undamaged data from the neutron diffraction. Cross sections of the undamaged and damaged material were then prepared for EBSD in an attempt to understand how the damage may have affected the grains in the bulk as compared to the damage mechanisms outlined by Martina Scapin (2013) and Bertarelli (2014). TEM specimens of these affected grains were prepared *via* Focused Ion Beam (FIB) to determine if any of the common characteristics such as black spot damage could be detected.

## Materials and Methods

### Synthesis

The Nd_2_Zr_2_O_7_ pyrochlore samples were fabricated by a wet chemical synthesis approach. 0.1 molar aqueous solutions of Nd(NO_3_)_3_ and ZrOCl_2_ were prepared, mixed and the hydroxides were co-precipitated in 25% liquid NH_4_OH. A detailed description can be found elsewhere (Finkeldei et al., 2014). The dried and ground precipitate was calcined at 600°C for 2 h in air. The calcined powder was ground with a pestle and mortar and pellets were cold pressed with 40 kN in a 10 mm die for 7 s. Sintering of the pellets took place for 15 h at 1,600°C in air with a heating and cooling rate of 5.3°C/min. Since there were no similar starting materials available for titanium at the time the Er_2_Ti_2_O_7_ pellets were fabricated *via* a solid-state reaction procedure. Therefore, Er_2_O_3_ and TiO_2_ were mixed in the appropriate amounts for an equimolar ratio of Er and Ti. The powders were ground and mixed for 2 × 30 min at 550 rpm with a planetary ball mill in a ZrO_2_ mixing bowl with ZrO_2_ grinding media. A short-wet milling step in acetone was applied at 400 rpm for 3 min and the grinding media were separated from the milled powder *via* sieving. The same pressing procedure as for the Nd_2_Zr_2_O_7_ pellets was applied. A first sintering took place in air for 15 h at 1,600°C with a second grinding step following the above-described procedure applied, and the dried powder repressed into pellets. A second sintering step at the same conditions as before was applied.

(NdEr)_2_(ZrTi)_2_O_7_ pellets were synthesized from Er_2_O_3_, TiO_2_, Nd_2_O_3_ and ZrO_2_ starting powders. Equimolar amounts of Nd, Er, Zr and Ti were mixed in a mortar, homogenized and milled as described for the Er_2_Ti_2_O_7_ pellet fabrication. A calcination step took place at 900°C for 2 h with a fast heating and cooling rate. After grinding the calcined powder was pressed into pellets and sintered as described above. Post sintering the pellets had stuck to the aluminum sintering plate on the bottom of the pellets. For each composition eight pellets were prepared that were approximately 4 mm in thickness.

### Density Determination and Microstructural Characterization

The microstructure and chemical composition of the pellets were analyzed with a Quanta 200F from FEI and an EDS system from EDAX. The pellet microstructures were determined prior to the irradiation experiments.

The sintered density was determined geometrically for all fabricated pellets. For one pellet of each composition the density was also determined by the Archimedes method, for full data see supplementary information.

### Sample Irradiation

Slices of approximately 150 microns were cut to provide enough material for the surfaces to be ground to be parallel from the topside of the pellets. Sequential polishing using 15, 3, and 1/10 µm diamond slurry on hard, nap-less polishing cloths was then performed to obtain samples of approximately 100–130 microns in thickness whilst removing any damage produced due the cutting process. It was difficult to produce a set of samples that were consistently 100 microns in thickness due to the fragility of the samples. The samples were then irradiated with He^2+^ ions perpendicular to the surface, at multiple single energies (2, 3, 4, 5, and 6 MeV) at a fluence of 1 × 10^16^ ions/cm^2^, in a vacuum of 10^–5 ^Pa, on the ANSTO STAR tandem accelerator, at the Centre for Accelerator Science (CAS). Considering the uncertainty of penetration depth due to channeling, void production and other processes this is equivalent to a total depth of approximately 20–25 microns and is discussed in detail in the SRIM calculation section. The average He^2+^ particle current on the sample surface was around 500 nA, and it was continuously monitored by integrating the sample charge, and periodically monitored by Faraday cup measurements. The samples were mounted on a large heat sink (Al) as compared to the sample size, using thermal-conductive paste, and based on previous calibration experiments we concluded that the irradiation was carried out at approximately room temperature. From previous calibration runs, it was estimated that the temperature of the samples increased rapidly in the first minutes of irradiation and stabilized after that for the rest of the irradiation, well below 60°C for the entire length of the runs.

### SRIM Simulations

Displacements per atom were calculated with SRIM/TRIM 2013 (Ziegler et al., 2010) by using the *Detailed Calculation with full Damage Cascades* mode for the Er_2_Ti_2_O_7_ and Nd_2_Zr_2_O_7_ pyrochlores. This mode of the Transport of Ions in Matter (TRIM) program calculates all collisional damage of the He^2+^ ions within the target material. The densities used in calculations were determined by the Archimedes method for a single pellet for each chemical composition and slices from multiple pellets were used for the He^2+^ irradiation experiment. The density variation for the remaining pellets, was determined by dividing the weight by the volume of the pellet ( $V=\pi r^{2}h$ ) and below 0.9%.

The displacement energy E_d_ values that were provided within the TRIM program for Er_2_Ti_2_O_7_ and Nd_2_Zr_2_O_7_ were 25 eV, for the A and B site cations, and 28 eV for the oxygen anions for both compositions. Many of these displacement energies are only assumptions and as experiments or simulations are performed the values are updated in the package. To further complicate matters the experimental stopping powers for heavy ions contain far more scatter than for light ions, hence there are larger errors for heavy ions, Be–U. This is the reason why it important to source more recent values and according to the literature (Sickafus et al., 2000b; Li et al., 2015) it is highly unlikely that the E_d_ value for oxygen is larger than for the cations within a pyrochlore. Therefore, these values were replaced with E_d_ values resulting from molecular dynamic (MD) simulations by Dong et al. (2017) for the Er_2_Ti_2_O_7_. Dong et al. report average E_d_ values for each sublattice in an Er_2_Ti_2_O_7_ sample to be 85 eV for Er^3+^, 197 eV for Ti^4+^, and 75 eV for O^2-^. For Nd_2_Zr_2_O_7_ the TRIM auto-populate E_d_ values were replaced with average values for the three crystallographic directions [100], [110], and [111] from Xiao et al. (2015) which resulted in E_d_ energies of 28.5 eV for Nd^3+^, 32.2 eV for Zr^4+^, and 11.6 eV for O^2-^. These threshold displacement energies were determined *via ab initio* MD by Xiao et al. (2015). The higher E_d_ values for the titanate pyrochlore compared to the zirconate pyrochlore can be explained by their calculation *via* MD simulation, whereas the E_d_ values for the zirconate pyrochlore were calculated *via* ab-initio MD simulations.

One example of inconstancies in modeling radiation damage *via* SRIM is the study of Wittmaack et al. entitled “Reliability of a popular simulation code for predicting sputtering yields of solids and ranges of low-energy ions” (Wittmaack, 2004) where low energy is considered to be anything below an accelerating voltage of 5 keV. They report that there were several discrepancies between modeling *via* SRIM and experimental data and that the low-energy electronic stopping powers of SRIM-2003 were found to be much too low. They also reported that there were variations between SRIM-2000 and SRIM-2003 due to the changes in the software to address some of these issues, thus the latest version of the software should always be used. However, with each iteration, comparisons still need to be performed to determine the reliability of the software to predict both dpa and damage depth.

Other publications have investigated the discrepancy between modeling and damage depth (Simeone et al., 2015) with the majority finding that damage occurred deeper into the sample than predicted by SRIM, again being attributed to issues with electronic stopping.

Considering all the assumptions and issues the reader should bear in mind that these simulations then are only used as a guide with regards to dpa and penetration depth and the variation and reliability of SRIM have long been discussed in the literature, however as it is freely available and used widely in the community it has become the standard.

### Synchrotron and Neutron Diffraction Data Collection

Synchrotron X-ray powder diffraction (SXRD) was performed at room temperature on the powder diffractometer at beamline BL-10 of the Australian Synchrotron (Wallwork et al., 2007). Data were collected over the angular range 4 < 2θ < 84.5°, using X-rays of wavelength 0.72797 Å, as determined by structural refinement of NIST SRM660b LaB_6_ standard diluted with diamond powder. Samples (∼0.1 mg) were housed in a 0.3-mm-diameter glass capillary, which were then rotated during the measurements. Data was obtained using a bank of 16 Mythen detectors, each of which covers 5° and collected for 3 min at each of the two detector positions, to avoid gaps in the data from the individual modules.

Neutron powder diffraction (NPD) experiments were performed at the high-resolution powder diffractometer Echidna (Avdeev and Hester, 2018) at ANSTO’s Australian Centre for Neutron Scattering (ACNS) facility at Lucas Heights on both the irradiated and unirradiated samples of Er_2_Ti_2_O_7_ and Nd_2_Zr_2_O_7_ under the same conditions as a previous examination of LaB_6_ standard that had been manufactured with enriched ^11^B. Diffraction patterns were acquired at room temperature over an angular range of 14.876° < 2θ < 162.00°, at intervals of 0.125°, using neutrons of wavelength 1.622 Å, Supplementary Figure S1. The Er_2_Ti_2_O_7_ sample broke apart due to exposure to the He^2+^ ion beam and had to be held together wrapped in Al foil to ensure the geometry was as close as possible to the undamaged sample.

The SXRD and NPD data were analysed by the Rietveld method using the TOPAS refinement program (Coelho, 2018). The instrumental function was determined with the strain/stress-free NIST LaB_6_ standard reference material SRM660b (Black et al., 2020). Once the pseudo-Voigt functions were determined they were used for all subsequent data analysis to ensure that all effects on the spectra were due to the sample.

### EBSD Method

The Pyrochlore samples were prepared for EBSD analysis by mounting them in cross section to allow examination of the damaged through to the undamaged region. Sequential polishing using 15, 3 and 1 µm diamond slurry on hard, nap-less polishing cloths, was followed by a final polish using colloidal silica on a neoprene type polishing cloth. Both polished sample surfaces were then coated with approximately 1.5 nm of platinum *via* an ion sputter coater to prevent charging. The samples were then mapped using a Zeiss Ultra Plus SEM coupled with an Oxford Instruments HKL Nordlys EBSD detector system at an accelerating voltage of 20 kV. The EBSD maps were generated using the HKL Channel 5 software. The entire thickness of the samples was mapped with a step size of 0.106 and 0.129 μm for the Zr and Ti samples respectively and determined. A further map of the Zr sample was then performed on select grains of interest at higher magnification to locate an area for TEM sample preparation using the FIB.

### FIB Method

The instrument used for the TEM sample preparation was a Zeiss Auriga 60 focused ion beam microscope. A 20 × 2 µm rectangular layer of protective platinum was first deposited above the area of the desired cross section to protect the surface from the ion beam.

To ensure that neither damage was introduced or recovered/reduced the following method was used to prepare the TEM specimens. Coarse milling of a trapezoidal shaped trench was performed on both sides of this platinum layer using Ga^+^ ions for milling, the trench was first milled at 30 kV 8 nA and then a rectangular shaped cut was done 1.5 degrees off axis using 30 kV 2 nA to form the final lamella shape with parallel sides before lifting out. A “u” cut was then made at a shallow tilt angle in order to free the bottom and sides of the lamella. The lamella was then lifted out *in situ* using the OmniProbe 200 nanomanipulator system and attached onto a TEM copper grid *via* platinum deposition for further thinning and polishing inside the FIB. The polishing process involved positioning the lamella 0.5 degrees off the axis of the ion beam on each side starting with a 30 kV 600 nA ion probe, followed by a 30 kV 120 pA ion probe. The off-axis position accounts for the ion beam shape to keep the sides of the lamella parallel whilst polishing.

Further thinning was then carried out with a 15 kV 80 pA ion probe at 3 degrees off axis to remove the damage created by the 30 kV gallium ions. A final polishing step was applied with a 5 kV 20 pA ion probe at 3° then a long polish in deposition mode at 2 kV 20 pA at six degrees off milling access to remove any further damage created by the higher energy gallium ions in the previous steps. For comparison Engelmann et al. (2003) studied single crystalline Si, which is very sensitive to sputter amorphization. In their paper they state the following “The thickness of the amorphization layer could not be reduced significantly by decreasing the Ga^+^ ion energy down to 10 keV, reducing the probe current to the lowest possible value of 1 pA or varying the sputter angle. A reduction of the amorphization depth down to 7 nm was only possible with a 5 keV Ga^+^ ion beam (the lowest ion energy attainable in the used FIB tool) and tilting the lamella to ±7°, subsequent ion milling of the FIB-cut TEM lamella with 3.5 keV Ar^+^ ions at a tilt angle of 15° resulted in a near-surface amorphization depth of 5–6 nm. This value is close to the near-surface amorphization of 4 nm that was measured on a classically prepared TEM sample with final 3.5 keV Ar^+^ ion milling. Using 1.5 keV Ar^+^ ions and tilting the sample by ± 20° the near-surface amorphization is between 2 and 3 nm Further to this point in their study of Si etched *via* Ga ions Mayer et al. (2007) found the following “Reduction of the amorphous layer thickness in a sidewall of Si as a function of Ga energy. The cross sections of FIB lamellae show that FIB milling at 88° incident angle and 30, 5, and 2 ion energy results in amorphous layers of ∼22, 2.5, and 0.5–1.5 nm thickness, respectively”, “The reduction of sidewall damage in Si at 2 keV polishing can produce TEM specimens which reveal sub-Ångström information”, “The advantage to using the FIB itself is that the beam may be focused, and hence the specimen can be imaged for exact placement of the final polishing window. Similar low energy FIB techniques have been used to prepare specimens for atom probe analysis, where results show that no deleterious ion mixing occurs”. To ensure that the damage layer was either small or not visible on either the edge or covering the whole lamella, high resolution EBSD was performed on the Nd_2_Zr_2_O_7_ sample, Supplementary Figure S2. The image clearly shows essentially crystalline material and an absence of an amorphous layer or a recrystallized layer covering the lamella of a single orientation. In addition there was the absence of an oriented alteration layer as shown can occur by Mayer et al. (2007). We therefore have assumed that any damage observed in the TEM is due to irradiation by the He^2+^ ions and not due to sample preparation.

The atomic structure of the irradiated samples was studied using transmission electron microscopy (TEM) on the FIB lamellae. TEM was performed on a JEOL 2200FS microscope, operated at an accelerating voltage of 200 keV. The TEM was equipped with a Gatan UltraScan1000, and Orius camera. Atomic lattice images and diffraction patterns were collected in bright field, imaging in dark field mode did not yield further details about the samples such as the evidence of bend contours.

## Results

### Microstructural Characterization

The SEM images Supplementary Figure S3 reveal the microstructure of the Nd_2_Zr_2_O_7_, Er_2_Ti_2_O_7_ and (NdEr)_2_(ZrTi)_2_O_7_ sintered pellets prior to irradiation. The Nd_2_Zr_2_O_7_ sample shows a heterogeneous surface with high and low density areas. The Er_2_Ti_2_O_7_ sample showed a dense microstructure with a homogeneous porosity. The (NdEr)_2_(ZrTi)_2_O_7_ revealed the presence of a second phase that was identified *via* EDS to consist mainly of aluminum and neodymium and most likely evolved during sintering *via* contamination of the pellet with the sintering crucible. Therefore, the solid solution sample was not utilized in the irradiation experiments as the presence of a second phase may have had unforeseen effects on the strain and stress in the sample after irradiation.

### Irradiation Calculations

Supplementary Figure S4 in the supplementary information shows the TRIM calculations for the overall dpa/depth values for the Nd_2_Zr_2_O_7_ and Er_2_Ti_2_O_7_ samples respectively. The dpa plots refer to the summation of the subsequent He^2+^ irradiations with 2, 3, 4, 5, and 6 MeV with a fluence of 1 × 10^16^ ions/cm^2^.

### Synchrotron XRD

The instrument was characterized with a standard composed of diamond and LaB_6_ (SRM 660b—Line Position and Line Shape Standard for Powder Diffraction) to provide instrumental parameters for Rietveld refinement, the background was estimated using a 16-term shifted Chebyschev function. The reasoning behind the use of LaB_6_ is that the material is produced to be strain free and so once instrumental parameters have been set any subsequent change in these parameters is due to the sample and not a variation of the instrument. Diamond has been added to the LaB_6_ to dilute the sample and thus reduce the issue of absorption from the Lanthanum in the sample which in this geometry may result in variation of intensity or position of the diffracted peaks. Scale factor, detector zero-point, lattice parameters, atomic coordinates and atomic displacement parameters were refined together with the peak profile parameters. Reasonable fits were obtained with the following values of R_wp_ 4.64%, R_p_ 3.32%, GOF 2.73. The peaks were fitted with pseudo-Voigt (PV_TCHZ) peak type (Thompson et al., 1987) with the following parameters, U = 0.000189(9), V = −0.000201(12), W = 0.000031(5), Z = 0, X = 0.0002(14), Y = 0 these were then used for the refinements of the Er_2_Ti_2_O_7_, Nd_2_Zr_2_O_7_ and (ErNd)_2_(TiZr)_2_O_7_ samples. Parameters of note were as follows; LaB_6_ *a* = 4.1533842(19) Å, and ε = 0.000076(2), wt% = 7.817(18); Diamond *a* = 3.5642611(17) Å, ε = 0.0000980(8), wt% = 92.183(18) %. For comparison the quoted lattice parameter is *a* = 4.156826 (8) Å in the NIST certificate a difference of 0.0034418 Å. These measurements confirmed the applied analysis since the NIST LaB_6_ standard is manufactured to have no strain. Results of the refinements are given in Table 1. Note all values are quoted in accordance with the IUCR recommendations for statistical descriptors in crystallography.

**TABLE 1:** Refined parameters for samples Er_2_Ti_2_O_7_, Nd_2_Zr_2_O_7_ and (ErNd)_2_(TiZr)_2_O_7_ from S-XRD data. Where *a* is the lattice parameter, *x* (O) is the value of the *48f* site, B*A* is the overall isotropic displacement parameter of the *A* cation*,* B*B* is the overall isotropic displacement parameter of the *B* cation*,* B (O1) is the overall isotropic displacement parameter of the oxygen at the O1 position, B(O2) is the overall isotropic displacement parameter of the oxygen at the O2 position, *A*-O(1) is the distance between the A cation and the oxygen at the O1 position, *A*-O(2) is the distance between the A cation and the oxygen at the O2 position, *B*-O(1) is the distance between the B cation and the oxygen at the O1 position, ε0 is the calculated strain, R_*p*_ is the R-pattern (background corrected)*,* R_*wp*_ is the R-weighted pattern (background corrected)*,* and GOF is the goodness of fit.

Sample	Er_2_Ti_2_O_7_	Nd_2_Zr_2_O_7_	(ErNd)_2_(TiZr)_2_O_7_
*a,* Å	10.07834(5)	10.68052(2)	10.325528(11)
*x* (O)	0.3316(2)	0.3339 (19)	0.3274(2)
B *A*, Å^2^	0.469(3)	0.599(3)	Nd + 3 0.11(4)
			Er + 3 0.89(4)
B *B*, Å^2^	0.255(8)	0.456(5)	Zr + 4 0.730(13)
			Ti + 4 0.270(13)
B (O1), Å^2^	0.71(5)	1.15(4)	0.45(9)
B(O2), Å^2^	0.66(8)	0.81(7)	0.10(12)
*A*-O(1), Å	2.18201	2.3124	2.23554
*A*-O(2), Å	2.4609(14)	2.5905(11)	2.5509(18)
*B*-O(1), Å	1.9620(9)	2.0901(7)	1.9927(11)
ε0	0.000094(5)	0.0000938(10)	0.000232(3)
R_*p*_ *, %*	2.87	3.46	2.57
R_*wp*_ *, %*	4.3	4.76	4.57
GOF	1.95	2.54	2.95

**FIGURE 1:**
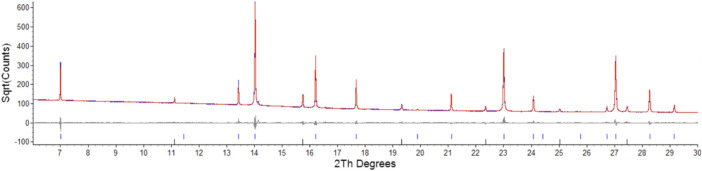
Low angle section of rietveld plot for the (ErNd)_2_(TiZr)_2_O_7_ sample, the red line is data, blue is calculated, grey is the difference plot, blue markers are the main phase and black markers are the unknown phase. Y axis is square root of counts to highlight the second phase, barely visible in a plot of counts vs. angle.

Parameters for Er_2_Ti_2_O_7_, Nd_2_Zr_2_O_7_ are consistent with those found in the literature (Farmer et al., 2014; Xu et al., 2015) and both refinements yielded strain results that are of the same order of magnitude as that of the LaB_6_ sample, therefore it can be assumed that the initial strain for both of these samples is zero. Refinement of the A and B site occupancy of the (ErNd)_2_(TiZr)_2_O_7_ solid solution sample indicates that the majority cation on the A site is Er and the majority on the B site is Zr with a stoichiometry of (Er_0.89_Nd_0.11_)_2_(Zr_0.73_Ti_0.27_)_2_O_7_. The diffraction pattern showed evidence of a second phase as shown in Figure 1, after matching of the six peaks present and based on the composition a compatible cif file was identified and it is most likely a cubic phase of space group $Pm\bar{3}m$ with a lattice parameter of 3.75670(3) Å. This cif file was used in the subsequent refinement and semiquantitative analysis. A structural analysis of the second phase was attempted but due to the scarcity of peaks and their low intensity a suitable refinement could not be obtained.

Semi-quantitative analysis resulted in values of 94.93(3)% for (Er_0.89_Nd_0.11_)_2_(Zr_0.73_Ti_0.27_)_2_O_7_ and 5.07(3)% for the unknown cubic phase. The (Er_0.89_Nd_0.11_)_2_(Zr_0.73_Ti_0.27_)_2_O_7_ lattice parameter lay between the Er_2_Ti_2_O_7_ and Nd_2_Zr_2_O_7_ samples at a value of 10.325528(11) Å. Moreover, contrary to the pure endmembers the solid solution sample contained strain at a value of 0.000232(3). Given the sample did contain inherent strain it would have been valuable to see the effect of radiation damage, however due to the presence of a second phase which may affect the results this sample was not included in the irradiation experiments.

### Neutron Diffraction

Defect fluorite as defined by Heremans et al. (1995) in their neutron diffraction study of Y_2_(Zr_*y*_Ti_1-*y*_)_2_O_7_ and often cited for example by Ewing et al. (2004), is described as follows, “Pyrochlore is an unusual oxide in that the order–disorder transformation occurs simultaneously on both the cation, as well as the anion lattice among three anion sites: 48f, 8a, and 8b. However, the cation and anion disordering may occur to different degrees and at different temperatures.” As stated by Ewing et al. (2004), Heremans et al. (1995) investigated the chemically induced order–disorder transition in the Y_2_(Zr_*y*_Ti_1-*y*_)_2_O_7_ system by analyzing the fractional occupancy of the interstitial 8a site and the effective scattering length for the A- and B-site cations, a measure of the extent of cation antisite disorder. With the increasing concentrations of Zr at the B-site, the structure of the Y_2_(Zr_*y*_Ti_1-*y*_)_2_O_7_ solid solution progressively changes to a defect-fluorite structure at *y* = 0.9. The anion disorder precedes the disordering of the cation lattice. The interstitial 8a site was filled immediately with the oxygen ions displaced from the nearest-neighbor anion site, the 48f oxygen, upon the addition of the larger Zr-cation. The occupancy of the interstitial 8a site increases linearly with Zr-content over the entire range of the solid solution. The onset of cation disorder occurred at *y* > 0.45 and was coupled with disordering of anions at the 8b site. Complete mixing of all three cation species occurs abruptly in the compositional range of 0.6 < *y* ≤ 0.9. The positional parameter for 48f oxygen has been found to increase sharply to 0.375 for the ideal fluorite structure due to the occupancy of oxygen at the interstitial 8a site and the decreasing average ionic radius difference at the A- and B-sites as the extent of cation mixing increases. In greater detail Heremans et al. (1995) report that for *y* = 0.3, 0.45, 0.6 and 0.9, the value of *x* for O(1) placed at 48f progressively reduced in the following sequence, 0.4173(2), 0.4110(2), 0.4022(3) and finally 0.375 with a corresponding increase in lattice parameter for *y* = 0.3, 0.45, 0.6 with *a*(Å) increasing from 10.1906(2), 10.2447(2) to 10.2890(5). We therefore have a set of common parameters to define if the irradiated pyrochlores are transforming to defect fluorite *via* examination of the O(1) position and a lattice parameter change, all of which can be provided by neutron diffraction.

The methodology used for the neutron diffraction data reduction was the same as that used for the synchrotron data to enable calculation of strain in the damaged samples.

The fit obtained for the LaB_6_ (SRM 660b—Line Position and Line Shape Standard for Powder Diffraction) had the following values of R_wp_ 9.22, R_p_ 6.98, GOF 1.41. The peaks were fitted with a pseudo-Voigt (PV_TCHZ) peak type of the following parameters, U = −0.03(8), V = −0.06(3), W = 0.24(3), Z = 0, X = 0, Y = 0 these were then used for the refinements of the Er_2_Ti_2_O_7_, Nd_2_Zr_2_O_7_ samples. The lattice parameter was *a* = 4.15644(7) Å as compared to 4.156826 (8) Å as quoted in the NIST certificate (a difference of 0.000379 Å) and the strain was effectively zero with a value of ε = 0.000(9). Results of the refinements are given in Table 2. Since there is a direct correlation between the occupancy of the sites and the overall isotropic displacement parameter the occupancy was not refined and set at a value of 1. If the occupancy was refined, a slight variation in this value may have resulted in an apparent variation in the overall isotropic displacement parameter and so give an indication of disorder, when there was none. The plot of the refined Nd_2_Zr_2_O_7_ sample demonstrating the quality of fit for the undamaged neutron data is given in supplementary Supplementary Figure S5.

**TABLE 2:** Refined parameters for neutron data. Where *a* is the lattice parameter, *x* (O) is the value of the *48f* site, B*A* is the overall isotropic displacement parameter of the *A* cation*,* B*B* is the overall isotropic displacement parameter of the *B* cation*,* B (O1) is the overall isotropic displacement parameter of the oxygen at the O1 position, B(O2) is the overall isotropic displacement parameter of the oxygen at the O2 position, *A*-O(1) is the distance between the A cation and the oxygen at the O1 position, *A*-O(2) is the distance between the A cation and the oxygen at the O2 position, *B*-O(1) is the distance between the B cation and the oxygen at the O1 position, ε0 is the calculated strain, R_*p*_ is the R-pattern (background corrected)*,* R_*wp*_ is the R-weighted pattern (background corrected)*,* and GOF is the goodness of fit. For the zirconate sample two pyrochlore phases were identified as consequence of irradiation which are both provided here.

Sample	Er_2_Ti_2_O_7_	Er_2_Ti_2_O_7_ (ir)	Nd_2_Zr_2_O_7_	Nd_2_Zr_2_O_7_ p1	Nd_2_Zr_2_O_7_ p2
*a,* Å	10.0878(18)	10.0906 (23)	10.6471 (18)	10.6780(3)	10.6443(4)
*x* (O)	0.4205(3)	0.4216(4)	0.3374(3)	0.3323(8)	0.3408(10)
B *A*, Å^2^	1.14(10)	0.96(17)	0.63(8)	1.0(3)	0.5(3)
B *B*, Å^2^	1.42(15)	1.6(2)	0.90(8)	1.3(3)	0.9(4)
B (O1), Å^2^	1.03(9)	0.80(15)	1.37(8)	1.8(3)	1.1(3)
B(O2), Å^2^	0.43(12)	0.30(19)	0.67(16)	0.1(3)	2.4(8)
*A*-O(1), Å	2.1841	2.1847	2.3052	2.3118	2.3046
*A*-O(2), Å	2.4777(18)	2.485(2)	2.557(2)	2.597(5)	2.531(6)
*B*-O(1), Å	1.9553(11)	1.9519(15)	2.0997(14)	2.085(3)	2.116(5)
R_*p*_ *, %*	2.98	2.97	2.54	2.81	2.81
R_*wp*_ *, %*	3.75	4.1	3.25	3.53	3.53
GOF	0.8	1.7	1.27	0.79	0.79

**FIGURE 2:**
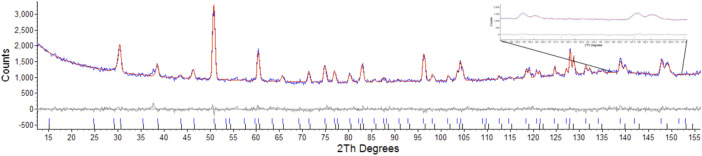
Rietveld refinement of radiation damaged Nd_2_Zr_2_O_7_ showing difference plot and assignment of both phases, insert highlights the two sets of peaks corresponding to the two pyrochlore phases.

Considering the conditions listed above to classify a pyrochlore transformation to a defect fluorite we can see from Table 2 the values for the Er_2_Ti_2_O_7_ sample do not vary greatly between the unirradiated and irradiated sample with regards to lattice parameter and the value of *x* at the 48f site. The Nd_2_Zr_2_O_7_ sample showed significant changes post irradiation with the neutron diffraction pattern showing evidence of two phases being present in the ratio of 57(4) % for phase 1 (p1) and 43(4) % for phase two (p2) when refined as two separate pyrochlore phases. The diffraction pattern showing two phases, difference plot and inset of the high angle peaks is given in Figure 2. The values are higher than expected for the irradiated volume and we will discuss this further on.

**FIGURE 3:**
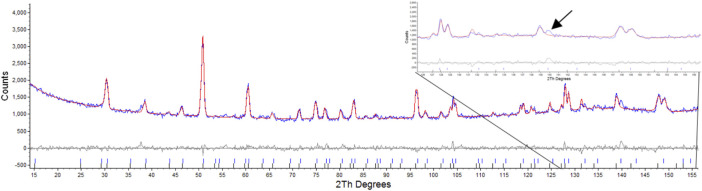
Rietveld refinement of radiation damaged Nd_2_Zr_2_O_7_ showing difference plot and assignment of defect fluorite and pyrochlore, insert highlights the absence of the equivalent reflections for the (10 6 4) and (12 2 2) that result only due to scattering from Oxygens as per Heremans et al.

Attempts were made to refine one phase as a defect fluorite as outlined by Heremans et al. (1995). This resulted in values of Rwp: 3.86, Rp: 3.04, for the pyrochlore phase and Rwp: 27.54, Rp: 38.37 for the defect fluorite phase, this was primarily due to the absences of peaks in model that result from scattering of the anions as outlined by Heremans et al. where for *hkl h* ≠ 4*n*, *k* ≠ 4*n*, and *l* ≠ 4*n*, *h* + *k* + *l* = 4*n* (Heremans et al., 1995) as shown in Figure 3. Attempts to refine the second phase with the reported defect fluorite structure for Nd_2_Zr_2_O_7_ with a lattice parameter of 5.1485Å and space group $Fm\bar{3}m$ (Loong et al., 1995) resulted in very high R factors (Rwp: 31.47, Rp: 40.86) and no further attempts were made with regards to this strategy.

Table 2 shows that both phases differ from the unirradiated sample, in that they have different overall isotropic displacement parameters in comparison to the unirradiated sample. Of interest is the fact that one of the phases has varied from the original lattice parameters by only 0.03% whereas the other varies by 0.3%, a difference of one order of magnitude in variation and like that reported by Heremans et al. however, both phases were refined to be pyrochlore. This is contrary to most reports in relation to irradiation of this phase that report the disappearance of the pyrochlore superstructure peaks indicating the transition to a defect fluorite, though most of these studies have been performed with X-ray diffraction which is less sensitive to Oxygen positions. To determine if the irradiation had resulted in positional disorder of either the cations, anions or both as outlined by Heremans et al. the refinements were repeated (Table 3) to determine the anisotropic displacement parameters for indications of initial movement of either cations or anions towards a fluorite like phase.

**TABLE 3:** Refined parameters where *A* U (1,1) and *A* U (1,2) are the anisotropic displacement parameters of the *A* cation*, B* U (1,1) and *B* U (1,2) are the anisotropic displacement parameters of the *B* cation*,* O1 U (1,1) and O1 U (1,2) are the anisotropic displacement parameters of the oxygen at the O1 position, O2 U (1,1) is the anisotropic displacement parameter of the oxygen at the O2 position, *A*-BVS is the bond valence sum of the A cation, *B*-BVS is the bond valence sum of the B cation, ε0 is the calculated strain, R_*p*_ is the R-pattern (background corrected), R_*wp*_ is the R-weighted pattern (background corrected), and GOF is the goodness of fit for the unirradiated and irradiated Er_2_Ti_2_O_7_ and Nd_2_Zr_2_O_7_ samples.

Sample	Er_2_Ti_2_O_7_	Er_2_Ti_2_O_7_ (ir)	Nd_2_Zr_2_O_7_	Nd_2_Zr_2_O_7_ p1	Nd_2_Zr_2_O_7_ p2
*A* U (1,1), Å^2^	0.0157(13)	0.0158(15)	0.0083(11)	0.015(3)	0.002(4)
*A* U (1,2), Å^2^	−0.0041(12)	−0.0040(13)	0.0011(11)	0.002(2)	0.010(4)
*B* U (1,1), Å^2^	0.018(2)	0.020(2)	0.0126(11)	0.019(4)	0.013(5)
*B* U (1,2), Å^2^	−0.006(3)	−0.006(3)	0.0077(17)	0.010(4)	−0.001(5)
O1 U (1,1), Å^2^	0.0123(11)	0.0125(12)	0.0214(13)	0.028(4)	0.017(4)
O1 U (1,2), Å^2^	0, 0, 0.0038(17)	0, 0, −0.0019(19)	0, 0, −0.0003(15)	0, 0, 0.009(4)	0, 0, −0.011(4)
O2 U (1,1), Å^2^	0.0075(19)	0.006(2)	0.001(2)	0.001(4)	0.021(10)
*A*-BVS	2.957(16)	2.950(16)	3.010(16)	2.821(38)	3.087(47)
*B*-BVS	4.087(13)	4.087(14)	3.893(17)	4.022(33)	3.800(41)
ε0	0.00(8)	0.000(3)	0.00(8)	0.0(3)	0.000(5)
R_*p*_ *, %*	2.98	2.97	2.54	2.81	2.81
R_*wp*_ *, %*	3.75	4.1	3.25	3.53	3.53
GOF	0.8	1.7	1.27	0.79	0.79

As before there is little difference between the irradiated and unirradiated Er_2_Ti_2_O_7_ sample with BVS values indicating that there is insignificant displacement of the cations or anions from their original ideal positions. It is highly likely that the irradiated region in the material has become amorphous and a comparison of diffraction patterns before and after irradiation on similar amounts of material shows evidence of an increase in background which can most likely be attributed to the scattering from the amorphous material. To determine the amorphous content, the neutron diffraction data from the irradiated sample was used to replace the data in the fit of the unirradiated sample and the background was not refined. A peaks phase was then inserted with several fundamental parameter peaks throughout the pattern and this phase refined to account for the additional incoherent scattering in the pattern. By inserting a peak phase, the chemical composition remained the same resulting in the same neutron scattering factors. The amount of amorphous material was then simply a percentage comparison of areas which were 17479 for the crystalline compared to 41488 for the amorphous, equivalent to approximately a 30% crystalline volume. If we consider the SRIM calculations indicated a minimum penetration of 20 microns into a 100–130 micron thick sample equating to 20–15% which is approximately an error of 1/3 and the potential errors in the penetration depth listed above they do not account for the variability. As stated previously the Er_2_Ti_2_O_7_ sample broke apart due to exposure to the He^2+^ ion beam and had to be held together wrapped in Al foil to ensure the geometry was as close as possible to the undamaged sample. However, if this was not the case and the sample was not in the center of the diffractometer due to this arrangement it is possible that this contributed to incoherent scattering, which was not accounted for in the method, i.e. the increase in background was attributed to amorphous material and not the incoherent scatter and so giving rise to a larger than expected value for amorphous content. Conversely, since thin Al foil was bent and wrapped around the sample the assumption was that it may exhibit strain which could be evaluated. Not only could the Al structure be refined but calculating strain by the same peak shape analysis method resulted in a value of 0.0004(14). This supports the applied methodology given a value of strain could not be determined for the ceramic component of the diffraction pattern due to a change in peak shape. Comparison of refined data for the Nd_2_Zr_2_O_7_ sample indicates that both phases 1 and 2 are different to the unirradiated sample. Variations can be seen in the displacement parameters for both the cations and the anions as compared to the undamaged sample. The calculated BVS indicated that there is over bonding and under bonding in each of the phases compared to the unirradiated specimen, however this is minimal. If A and B site mixing were occurring in phase 1 the BVS sums would not match so closely to the original sample, as compared to the work of Simeone et al. (2017) where they demonstrated an equivalent BVS of approximately 4.2 for La^3+^ and 3 for Zr ^4^+ for defect fluorite. If we consider that Heremans et al. reported approximately a 0.5% change in lattice parameter and given the irradiated phase exhibited a change of 0.3% one might expect a corresponding change in the x value of the 48f position in the oxygen. For this change in lattice parameter Heremans et al. reported a corresponding reduction of 1.5% in the value for 48f towards 0.375, our study also observed a 1.5% reduction from a value of 0.3374(3), thus moving the oxygen further from the ideal value of 0.375 not towards it. It is difficult to directly compare displacement parameters to the study of Heremans et al. because they have chosen to refine both the occupancy of the site with the overall isotropic displacement parameters whereas we have fixed ours at occupancy 1. There is an increase in disorder of the A and B cation and to highlight the possible difference in phases for the irradiated zirconate sample Supplementary Figure S6 shows the difference in the ellipsoids due to the anisotropic displacement parameters. Compared to the unirradiated sample, phase 1 shows evidence of the Nd and Zr cations increasing in disorder parallel with the 111 plane while the O1 oxygens have moved slightly in a direction perpendicular to the 111 plane. In comparison phase 2 indicates a reduction in disorder of the cations while the O1 anions are now being stretched out parallel with the 111 plane. It was anticipated that analysis of the neutron diffraction data would indicate a migration of cations and anions towards an equivalent fluorite structure as reported but a comparison from previous indicates that this is not the case if we remember the statement “The anion disorder precedes the disordering of the cation lattice. The interstitial 8a site was filled immediately with the oxygen ions displaced from the nearest-neighbor anion site, the 48f oxygen, upon the addition of the larger Zr-cation”. In the same paragraph Ewing et al. (2004), made the contradictory statement “Pyrochlore is an unusual oxide in that the order–disorder transformation occurs simultaneously on both the cation, as well as the anion lattice among three anion sites: 48f, 8a, and 8b. However, the cation and anion disordering may occur to different degrees and at different temperatures.” This can be interpreted as a contradiction given the statements; the anion disorder first occurs, that cation disorder can occur simultaneously and that it can occur to different degrees at different temperatures. The standard definition often seen in the literature for the presence of defect fluorite is the loss of superlattice reflections in a powder diffraction pattern; in our analysis thus far, we have shown that the superlattice reflections are maintained, the oxygen at position (O1) does not move towards a value of 0.375 and that there is very little if any evidence of A and B site mixing due to bond valence sum calculations and yet there is a significant lattice parameter shift. Please note we have provided the raw neutron diffraction data as excel files in the supplementary information for the reader to examine.

Given that the Rietveld analysis did not indicate the presence of strain in either of the two pyrochlore phases that evolved from the irradiation of the zirconate sample and yet phase 1 accounted for approximately 50% of the coherent scattering pattern (much larger than that predicted by SRIM) as determined by quantitative phase analysis, one possible explanation for the appearance of the second phase is that a significant portion of the sample is in a stressed state. One could argue that the second phase is due to internal gas pressure however if we consider the work of Agarwal (2015) who demonstrated that He^2+^ is essentially insoluble in pyrochlore it is unlikely that we can attribute the total effect due to He^2+^. Drawing upon the work of Martina Scapin (2013), Bertarelli (2014), Oden (1992) who studied residual stress in ceramics where they demonstrated that for the case of Cu irradiation of Al_2_O_3_ as the penetration depth increases the surface effect will have a limited influence and a hydrostatic stress state is expected, we will assume the second phase to be a region of hydrostatic stress where all three strains are equal. If we consider that the Er_2_Ti_2_O_7_ broke apart due to expose to the He^2+^ it is highly likely that are large degree of swelling has occurred withing the interaction region and it has failed due to hydrostatic stress. To investigate further the role hydrostatic stress could play, we have assumed the amount of stress in the damaged sample where the average lattice strain in any given direction [*hkl*] can be defined by:

$$\varepsilon_{hkl}\;=\;\frac{d-d_{0}}{d_{0}}$$

Where *d* is the stressed lattice spacing and *d*_*0*_ is the stress-free lattice spacing along the respective [*hkl*]. Given that no peak broadening could be determined which would be indicative of strain, it is possible that the values were too small, a condition commonly associated with hydrostatic or volumetric stress. Examination of the differences in d-spacing between the non-irradiated and irradiated samples showed a uniform change, that is the value of *d*-*d*_0_ was consistent for each reflection as can been seen in the spreadsheets provided in the supplementary information. If this value did vary it could be an indicator that stacking faults were present. For example, in a cubic system where say the value of *d*-*d*_0_ for the 220 reflection (h ± k ± l ≠ 3n) as compared to say the 111 and 222 reflections is different this can indicate the presence of a stacking fault and reveal its orientation (Dupraz et al., 2015). As per Bartolomé et al. (2008) where they found that the average strains in zirconia toughened alumina were essentially equal leading them to conclude that the stress was hydrostatic and that the stress normal to the surface may be approaching zero due to the apparent depth as per Debelle and Declémy (2010) and Oden (1992). The deviatoric stress and strain based on Hooke’s Law can be represented as follows per (Noyan and Cohen, 1987), where $\epsilon_{ij}$ is the strain tensor, *E* is Young’s modulus, ν is Poisson’s ratio, $\sigma_{ij}$ is the stress tensor, $\delta_{ij}$ is the Kronecker delta, and $\sigma_{kk}$ is the average stress:

$$\epsilon_{ij}=\;\frac{1}{E}\left[\left(1+\upsilon \right)\sigma_{ij}-\upsilon \delta_{ij}\sigma_{kk}\right]$$

If we multiply both sides of the above equation by $\delta_{ij}$ the net result is:

$$\epsilon_{kk}=\;\frac{\left(1-2\upsilon \right)}{E}\;\sigma_{kk}$$

where $\epsilon_{kk}$ is the hydrostatic strain. The hydrostatic strain is simply the average of the three normal strains of any strain tensor. It should also be noted that one confusing aspect of hydrostatic strain is that it can be nonzero in incompressible materials such as ceramics. It is the determinant of the deformation gradient that is the true measure of volume change, and hydrostatic strain is only a convenient approximation of that when the strains are small. This is the case for our example where the observed strain for the (111) is approximately 0.00289; where the definition of small strain refers to the situation where we assume that changes after a displacement are so small that the total geometry is virtually unchanged.

If we now consider the above equation as follows, where the hydrostatic strains are now represented as $\bar{\varepsilon }$ (the average strain) and the hydrostatic stress σ :

$$\sigma =\frac{E}{1-2\nu }\;\bar{\varepsilon }$$

The above equation allows us to determine the value and nature of the hydrostatic stress for the two phases seen in the radiation damaged pyrochlore by the use of the published parameters for Young’s modulus and Poisson’s ratio by Feng et al. (2011) which are 219 GPa and 0.222 respectively, yielding values of 1.141 MPa for phase 1 and -0.105 MPa for phase 2. These values indicate that phase 1 is under tensile stress and that phase 2 is under a very small amount of compressive stress.

To examine this concept further recounting as per Sattonnay et al. (2007) if the material is elastically isotropic, only two elastic constants, the Young modulus and the Poisson ratio, are required to describe the elastic behavior of the material in any state of stress. We can take advantage of the reported elastic coefficients of Nd_2_Zr_2_O_7_ (Feng et al., 2011), which are C_11_ = 243 GPa, C_12_ = 69 GPa, and C_44_ = 47 GPa. For a cubic system the values for elastic compliance can be obtained from the coefficients *via* the following formulae.

$$S_{11}=\frac{C_{11}+C_{12}}{\left(C_{11}-C_{12}\right)\left(C_{11}+2C_{12}\right)}$$

$$S_{12}=\frac{-C_{12}}{\left(C_{11}-C_{12}\right)\left(C_{11}+2C_{12}\right)}$$

$$S_{44}=\frac{1}{C_{44}}$$

We therefore obtain compliance values of S_11_ = 0.00471 GPa, S_12_ = -0.00104 GPa and S_44_ = 0.02128 GPa for Nd_2_Zr_2_O_7_.

By employing the Voight-Reuss approximation (K. D. Verma and D. Nag, 1965) where the Voight bulk modulus (B_v_) and shear modulus (G_v_) are given by the following:

$$B_{v}=\frac{\left(C_{11}+2C_{12}\right)}{3}$$

$$G_{v}=\frac{\left(C_{11}-C_{12}+3C_{44}\right)}{5}$$

and the Reuss bulk modulus (B_r_) and Reuss shear modulus (G_r_) are

$$B_{r}=\frac{1}{3S_{11}+6S_{12}}$$

$$G_{r}=\frac{15}{4S_{11}-4S_{12}+3S_{44}}$$

For the coefficients of Nd_2_Zr_2_O_7_ we obtain Voight values of B_v_ = 127 GPa and G_v_ = 63 GPa which are consistent with the values of Feng et al., and Reuss values of B_r_ = 127 GPa and Gr = 172.77 GPa. The Voight and Reuss values represent the upper and lower limits of polycrystalline constraints, and the estimate of the bulk and shear moduli can be taken as the mean of the extremes. The elastic anisotropy can be described by the universal anisotropic index A^U^ (K. D. Verma and D. Nag, 1965) and the indexes of compression and shear are denoted by A_B_ (Rice, 1971) and A_G_ (Ravindran et al., 1998) all of which can be calculated as follows:

$$A^{U}=5\frac{G_{V}}{G_{R}}+\frac{B_{V}}{B_{R}}-6$$

$$A_{B}=\frac{B_{V}-B_{R}}{B_{V}+B_{R}}$$

$$A_{G}=\frac{G_{V}-G_{R}}{G_{V}+G_{R}}$$

For these three a value of zero indicates elastic isotropy and the variation from zero indicates anisotropic elastic properties. Our calculation resulted in values of A^U^ = −3.177 indicating anisotropic behaviour; A_B_ = 0 indicating isotropic compression and A_G_ = −0.4656 indicating the sample will exhibit shear anisotropy. To examine the effect of this we can calculate Young’s modulus in the normal direction of the three low index planes (100), (110), (111) (Zhang et al., 2007).

$$\frac{1}{E_{hkl}}=S_{11}-2S_{0}\frac{{\left(hk\right)}^{2}+{\left(hl\right)}^{2}+{\left(lk\right)}^{2}}{{\left(h^{2}+k^{2}+l^{2}\right)}^{2}}$$

Where,

$$S_{0}=S_{11}-S_{12}-\frac{1}{2}S_{44}$$

And Poisson’s ratio can be calculated for the three lower index planes in directions (*hkl*) in orthogonal directions (Zhang et al., 2007);

$$\upsilon \left(hkl,\theta \right)=\left\{S_{12}+\frac{S_{0}}{\left(h^{2}+k^{2}+l^{2}\right)}\left[{\left(\frac{h^{2}l}{\sqrt{h^{2}+k^{2}}\sqrt{h^{2}+k^{2}+l^{2}}}\cos\theta -\frac{hk}{\sqrt{h^{2}+k^{2}}}\sin\theta \right)}^{2}+{\left(\frac{k^{2}l}{\sqrt{h^{2}+k^{2}}\sqrt{h^{2}+k^{2}+l^{2}}}\cos\theta +\frac{hk}{\sqrt{h^{2}+k^{2}}}\sin\theta \right)}^{2}+{\left(\frac{l\sqrt{h^{2}+k^{2}}}{\sqrt{h^{2}+k^{2}+l^{2}}}\cos\theta \right)}^{2}\right]\right\}/\left[-S_{11}+2S_{0}\frac{{\left(hk\right)}^{2}+{\left(hl\right)}^{2}+{\left(lk\right)}^{2}}{{\left(h^{2}+k^{2}+l^{2}\right)}^{2}}\right]$$

the calculated values for Young’s modulus and Poisson’s ratio are given in Table 4.

**TABLE 4:** Young’s modulus in the normal direction of the three low index planes (100), (110), (111) and Poisson’s ratio can be calculated for the three lower index planes in directions (*hkl*) in orthogonal directions.

	Direction	$E_{hkl}$ GPa	υ(hkl,θ)	Stress p1 MPa	Stress p2 MPa
Plane (100)	[010]	212.4808	0.2211	1.1039	−0.1020
	[00 $\bar{1}$ ]	212.4808	0.2211	1.1039	−0.1020
Plane (110)	[ $\bar{1}$ 10]	139.8231	0.1455	0.5715	−0.0528
	[00 $\bar{1}$ ]	139.8231	0.4875	16.1793	−1.4947
Plane (111)	[ $\bar{1}$ 10]	125.5164	0.3353	1.1039	−0.1020
	[11 $\bar{2}$ ]	125.5164	0.3353	1.1039	−0.1020

There is no anisotropy in Bulk modulus with only a slight anisotropy in the Young’s modulus which is still comparable to the value of the Bulk modulus. Variations in Poisson’s ratio in two of the planes (100) and (111) does not occur however there are obvious anisotropic behaviour in the (110) plane as related to the shear anisotropy noted in the value of A_G._ This may appear large but if we compare these values to the system Ni_3_Al (Luan et al., 2018) which is an alloy but nonetheless can serve as an example of a material that does exhibit anisotropic behaviour and so will be subject to higher shear stresses, the values for Young’s modulus were 112.094, 212.973, and 304.241 Gpa for the (100), (110) and (111) planes, respectively and Poisson’s ratio in the (110) for the same directions were -0.14 and 0.76 respectively. The equivalent calculated values of stress for all directions except for the (110) plane in the [00 $\bar{1}$ ] are all very similar to the values obtained for the bulk which leads us to assume that the behaviour of Nd_2_Zr_2_O_7_ will be relatively uniform. This result reinforces the concept that for Nd_2_Zr_2_O_7_ the material is essentially elastically isotropic and only two elastic constants, the Young modulus and the Poisson ratio, are required to describe the elastic behavior of the material in any state of stress.

Therefore, the most likely explanation for the appearance of two phases post irradiation is that the sample has stress in the irradiated region and the variation in lattice parameters is due to hydrostatic tensile (p1) and compressive (p2) stress. To gain further insight into the irradiated regions and the concept that the incident ions had imparted a hydrostatic stress front past the predicted depth by SRIM the samples were then examined *via* EBSD and TEM.

### EBSD

**FIGURE 4:**
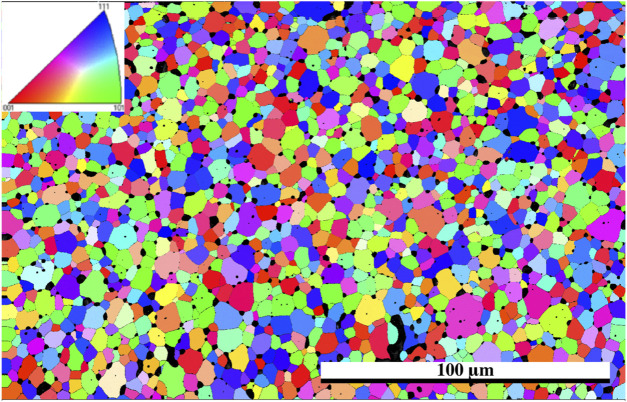
EBSD grain orientation map of Er_2_Ti_2_O_7_ utilizing cif generated from neutron diffraction of the unirradiated specimen, direction of He^2+^ ions are from the bottom of the image, inset shows pole figure orientation.

The EBSD map of the Er_2_Ti_2_O_7_ sample was generated based on the cif file of the unirradiated specimen obtained from neutron diffraction analysis. The purpose of performing EBSD was to determine if any gross changes had occurred from the damaged to the undamaged region and if there was a change in grain size and orientation as reported by Wen et al. (2016). As can be seen in the orientation map (Figure 4) there is no observable change from the damaged region at the bottom of the image to the undamaged region at the top. Given that the refinement of patterns indicated that only one phase was present in the sample that had been irradiated along with amorphous material, it is conceivable that in the damaged areas there is still sufficient coherence to generate a pattern for the EBSD to detect, giving the appearance of a still fully crystalline material. Considering that this study was at <1 dpa, the crystalline appearance of the only slightly damaged material in the EBSD pattern is understandable.

**FIGURE 5:**
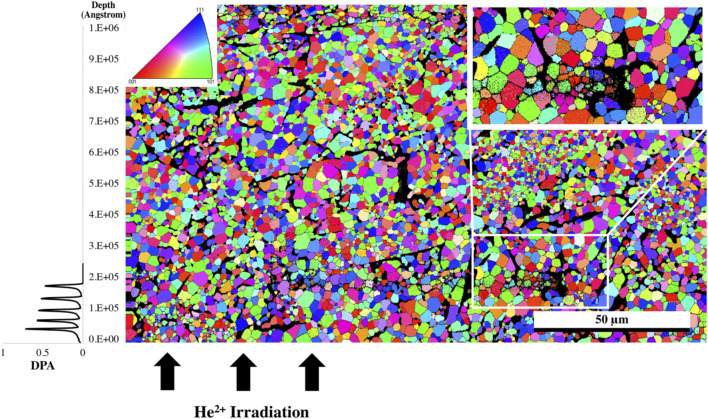
EBSD grain orientation map of the irradiated Nd_2_Zr_2_O_7_ sample utilizing the cif generated from neutron diffraction assigned to the undamaged part of the sample, direction of He^2+^ ions are from the bottom of the image. The white rectangle indicates the area where the high-resolution EBSD was taken in Figure 6, and the top left inset shows pole figure orientation. The plot to the left is the SRIM simulation and is provided as a comparison for penetration depth of the He^2+^ ions.

For comparison the Nd_2_Zr_2_O_7_ sample was mapped in the same way as the Er_2_Ti_2_O_7_ sample to determine if there was variation between the damaged and undamaged regions. For consistency the cif file that was assigned to the undamaged unstressed region was used for mapping. As can be seen in Figure 5 there is an overall greater variation in grain size which is inherent to the sample. However, there is a slight indication in the damaged region that some of the grains had broken up.

**FIGURE 6:**
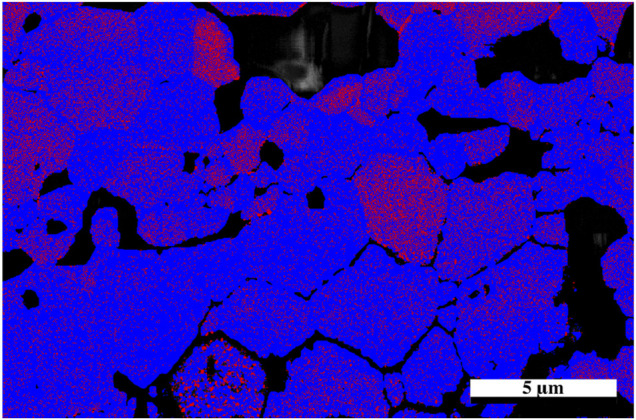
High resolution EBSD phase map of irradiated Nd_2_Zr_2_O_7_ utilizing cifs generated from neutron diffraction assigned to the undamaged and damaged parts of the sample, direction of He^2+^ ions are from the bottom of the image. Red areas indicate phase 1.

The sample was then mapped with the two neutron diffraction generated cif files: one associated with the undamaged region and the other from the damaged region associated with the residual stress (phase 1). The resultant map indicated a larger proportion of phase 1 in the damaged region and so a higher resolution map was taken of that area. It should also be noted with closer examination that there appears to be damaged grains deeper than the stated penetration by SRIM calculations which is consistent with a hydrostatic stress field penetrating beyond the predicted layer (Martina Scapin, 2013; Bertarelli, 2014) with some appearing as deep as 30 microns into the sample, however the center region of the sample appears to be damage free. The results from the high-resolution EBSD phase map Figure 6 reveal some grains that show very little of phase 1 while others appeared to contain a uniform distribution of phase 1 throughout the grain. It is possible that as the sample accumulates damaged regions, a buildup of residual stress will lead to grain refinement as seen in alloys thus causing the larger grain to break up into smaller grains. The fact that some grains are more damaged than others may also be an artifact of orientation and the damage occurring is related to the anisotropy as outlined in the diffraction results.

### TEM Analysis of the Irradiated Pyrochlore Specimens

#### Er_2_Ti_2_O_7_

**FIGURE 7:**
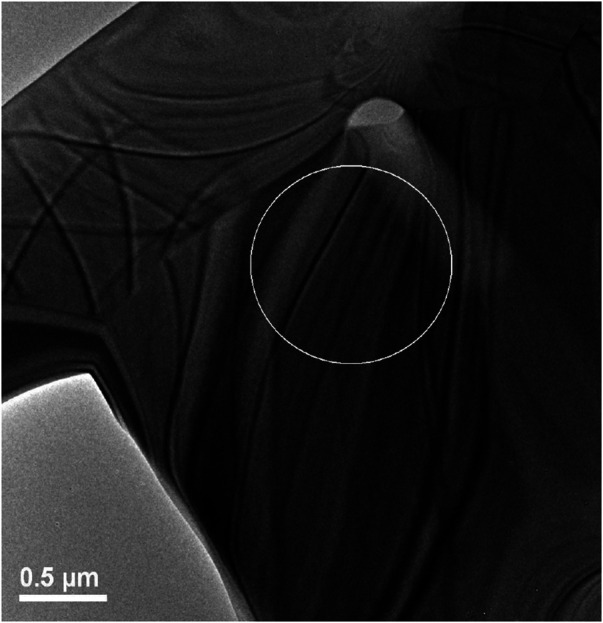
Image showing region where high resolution TEM was performed on irradiated Er_2_Ti_2_O_7_ sample. Grain boundaries can easily be seen in the image as well as bend contours.

**FIGURE 8:**
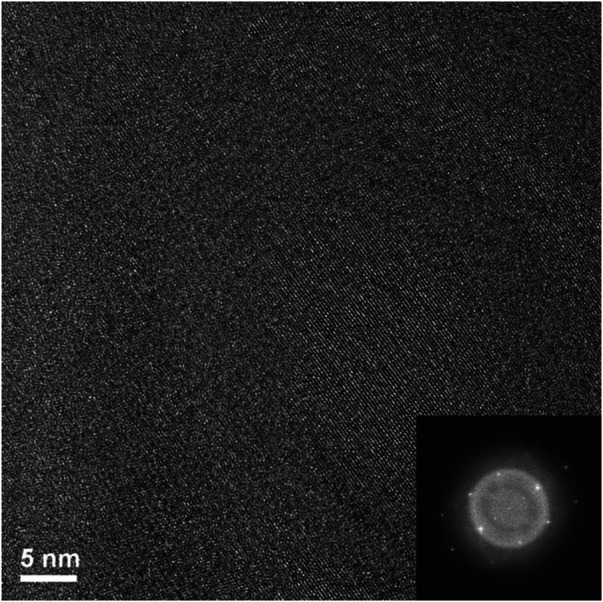
High resolution TEM image of circled area of irradiated Er_2_Ti_2_O_7_ from Figure 5 and the FFT of high-resolution image (inset).

A lower magnification bright field image of the TEM sample prepared by FIB methods is shown in Figure 7. Bend contours in this image indicate that the material is predominantly crystalline; however, this does not preclude the presence of disorder due to radiation damage. A high-resolution TEM image was recorded from a location within the circled area of Figure 7 and is shown in Figure 8.

By reference to the scale bars, the image shown in Figure 8 is much smaller than the circled area shown in Figure 7 and therefore may sit mostly within a band of lighter grey contrast within the circled area. Note that even though there are bend contours in Figure 7, the lighter bands are not necessarily tilted completely out of a diffracting condition due to distortion of the sample. Furthermore, the high-resolution image clearly shows the presence of lattice fringes that are consistent with the two strongest diffracted beams in the FFT, which is shown in the inset of Figure 8. This diffraction pattern is close to, but not precisely aligned on a [110] zone axis of pyrochlore, such that one of the two <111> systematic rows is excited more strongly than the other. The stronger row exhibits a pair of (444) beams, a pair of stronger (222) beams sitting on a diffuse ring, and a pair of very weak (111) beams inside of the diffuse ring. The measured spacing between the lattice fringes is approximately 0.3 nm, which is close to the spacing of the (222) lattice planes of Er_2_Ti_2_O_7_. The lattice image also shows some local areas wherein the fringes are less distinct or absent altogether and this is consistent with the diffuse scattering shown in the diffraction pattern. Similar electron diffraction patterns and images have been observed previously in pyrochlore and interpreted in terms of strain and domain size during the crystalline to amorphous transformation in pyrochlore (e.g., Lumpkin and Ewing, 1988).

**FIGURE 9:**
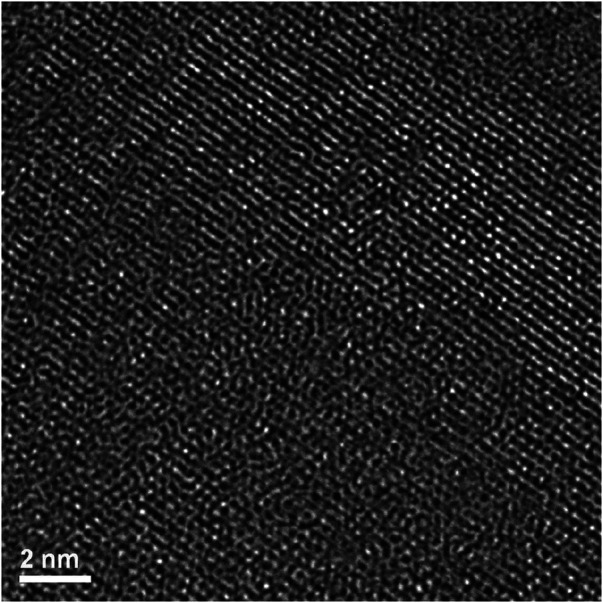
Magnified area of high-resolution region of irradiated Er_2_Ti_2_O_7_ showing ordered and disordered regions.

To further investigate the arrangement of disordered and ordered material in the irradiated region an image at a higher magnification but at the same orientation as Figure 8 was taken of the previous image, (Figure 9). There are clear regions of disorder and order, with some of the disorder existing within the areas where lattice fringes are visible and areas outside of the lattice fringes. As with Figure 8 this appearance is like former crystalline to amorphous transformation in pyrochlore studies (Lumpkin and Ewing, 1988; Lian et al., 2009).

#### Nd_2_Zr_2_O_7_

To investigate the possible cause of the dual phases visible in the high-resolution EBSD image (Figure 6) a TEM sample from the area highlighted in Figure 5 was prepared *via* FIB for high resolution TEM examination. Two regions were examined and are circled in the image to provide a reference to indicate where the presented images originate from, it should be noted that these areas were representative of the entire grains and the TEM image was recorded from a location within the circled area.

**FIGURE 10:**
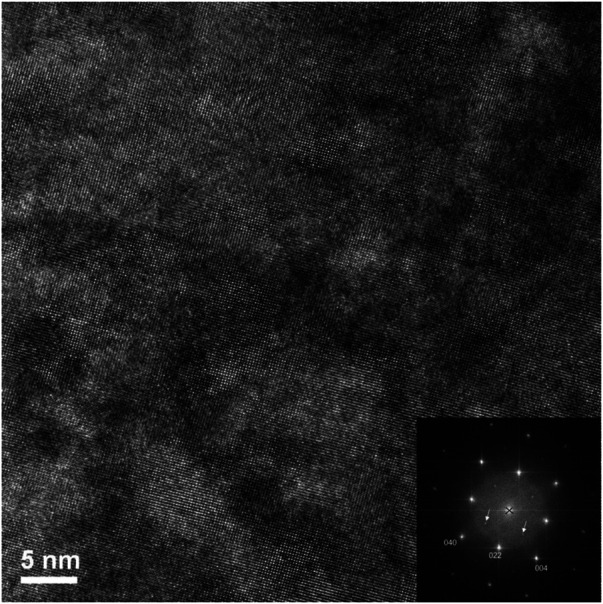
High magnification image of irradiated Nd_2_Zr_2_O_7_ area at the top of the lift out of Figure 12 showing no evidence of disorder.

Firstly, there are areas that appear to be very faint bend contours like those seen in the Er_2_Ti_2_O_7_ sample, therefore the sample was imaged in dark field mode and is given in Supplementary Figure S7. Imaging in objective aperture dark field mode highlights the presence of bend contours. However, the image was acquired in STEM mode with an annular dark field detector and the contrast mechanism is different, even so, if the crystal was bent to any degree, it should be visible and as can be seen in the supplementary figure there was no evidence of high contrast areas, an indication that the sample is bent. The area at the top of the sample that corresponded to a low abundance of secondary phase will be discussed first. As can be seen in the high-resolution image Figure 10, the area displays a very high degree of order (crystallinity), and lattice fringes are visible throughout most of the sample. A FFT of the image was performed (inset of Figure 10) and there was very little evidence of disorder, which was more pronounced for the Er_2_Ti_2_O_7_ sample, Figure 7.

**FIGURE 11:**
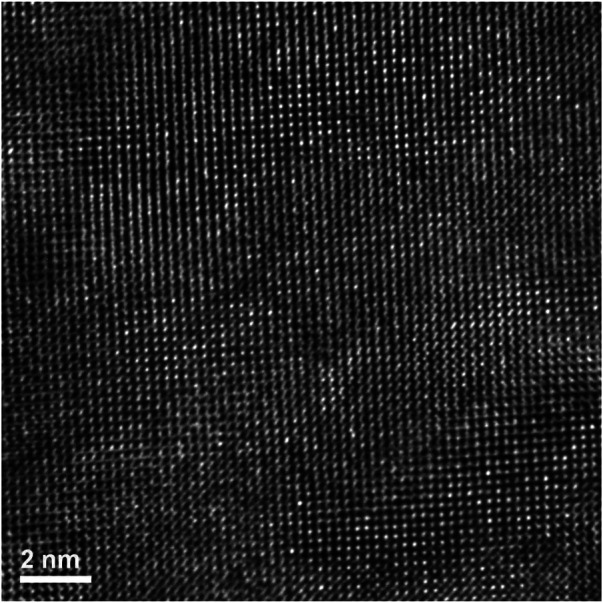
Lift out area of the irradiated Nd_2_Zr_2_O_7_ sample prepared *via* FIB. The two chosen areas contain a high and low amount of second phase of the altered pyrochlore phase after irradiation, respectively.

To determine if disorder was present within the lattice fringes as in the Er_2_Ti_2_O_7_ sample an image (Figure 11) was acquired at higher magnification at the center of Figure 10. It is apparent that the area examined is devoid of disorder with regular lattice fringes clearly visible.

**FIGURE 12:**
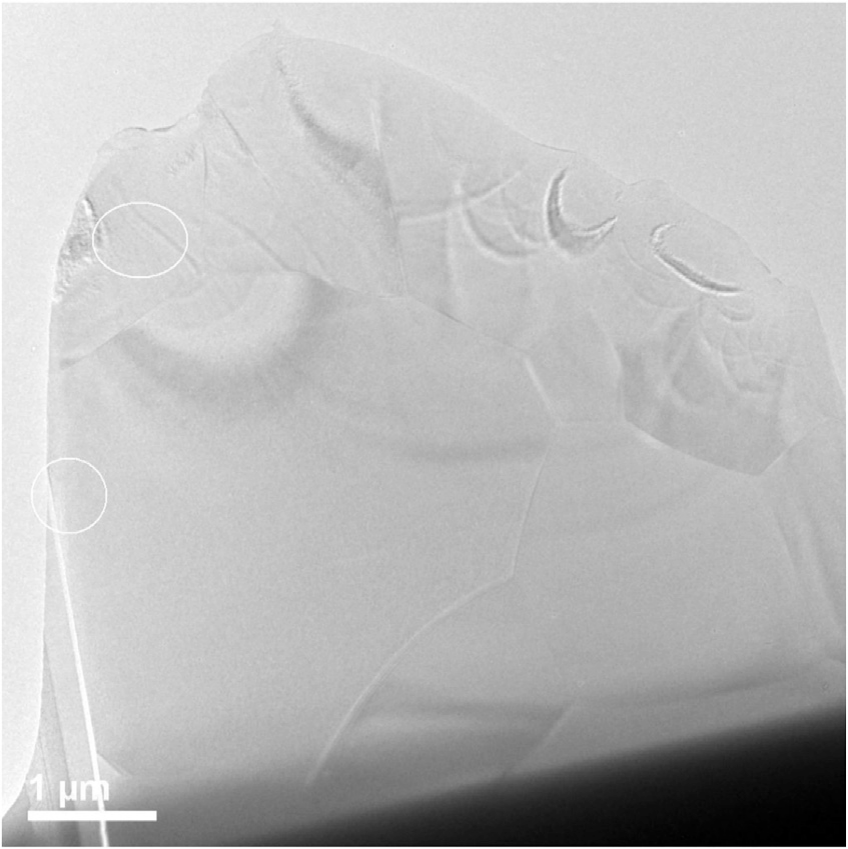
Top of lift out area in Figure, showing highly ordered lattice fringes. The FFT inset of the high-resolution image is viewed down the [1 0 0] and shows a very small amount of diffuse scattering, possibly from amorphous material. Superlattice reflections are indicated by the arrows.

**FIGURE 13:**
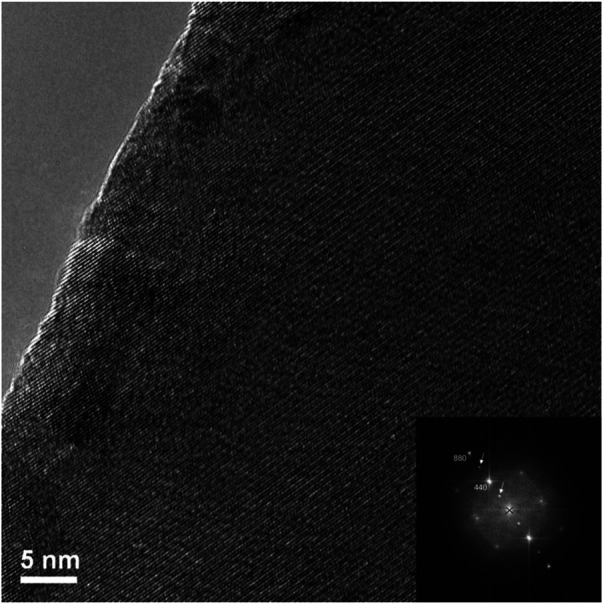
High resolution image of lower circled FIB lift out area of irradiated Nd_2_Zr_2_O_7_ (Figure 12) showing intertwined regions of disorder. The FFT inset of the high-resolution image is viewed down the [−1 −1 1], the diffuse scattering is indicative of the areas of disorder that can be seen in the image. Superlattice reflections are indicated by the arrows.

The region corresponding to a high proportion of the second phase (from a location within the circled area in Figure 12) was subsequently examined *via* high resolution TEM, Figure 13. This diffraction pattern is close to, but not precisely aligned on a [−1−11] zone axis of pyrochlore. There are obvious areas of disorder, however what is of interest is that they appear to be intertwined within the lattice fringes. This observation/behavior is very different to the Er_2_Ti_2_O_7_ sample where large areas of disorder were visible and may be mainly due to the difference in radiation tolerance as discussed in the introduction.

To confirm that these regions were in fact disordered or areas of amorphous material a FFT was performed on the image (inset). The FFT clearly exhibits characteristics associated with disorder evidenced *via* the diffuse scattering visible in the inset in Figure 13, With the diffuse ring sitting on the (440). A region of amorphous material as described by Mayer et al. (2007) was visible along some sections of the lamella as shown in Supplementary Figure S8.

**FIGURE 14:**
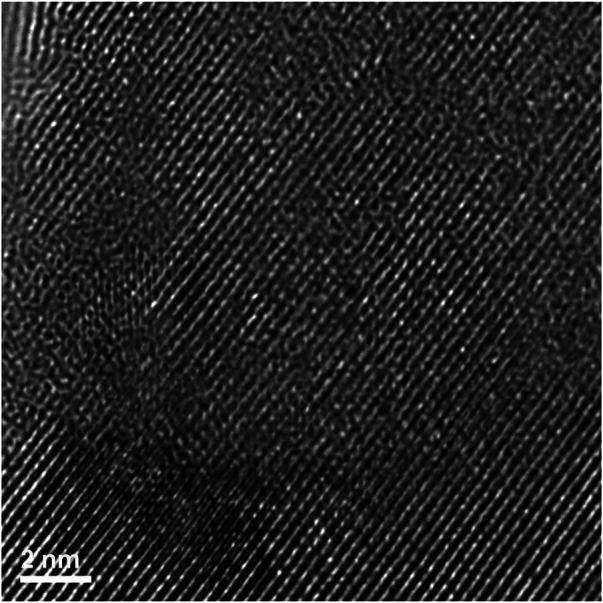
High magnification image showing how the disorder exists for irradiated Nd_2_Zr_2_O_7_ within the region where lattice fringes are visible.

A high magnification image (center of Figures 13, 14) shows how the disorder is intertwined throughout the sample. The change from order to disorder could be described as a continuum whereas the Er_2_Ti_2_O_7_ sample had regions that were either ordered or disordered, indicating a different radiation response mechanism for the titanate and zirconate pyrochlore samples. One issue with the thickness of the TEM lamellae is the effect of being able to resolve the high-resolution image to some depth, z, into the crystal which may also be causing distortion of the lattice fringes. The variation adjacent to the areas of damage may have some domains, possibly point defects and clusters that are a result of the adjacent damage. However these areas of damage are very similar to those seen in previous studies of Lumpkin and Ewing (1988) and Lian et al. (2009).

## Discussion

The aim of the study was to detect the early onset of phase transformation in relation to stress and strain of different pyrochlores, one prone to amorphization, one reported to undergo a transformation to defect fluorite and one a solid solution. With regards to the solid solution of the Zirconate and Titanate pyrochlore, the synthesis method employed was not sufficient to achieve a 50:50 ratio of A and B cations according to the diffraction results. However, the fact that the as fabricated sample contained strain is an indicator that if the sample could be synthesized to have a 50:50 ratio analysis of the irradiation response would be insightful regarding whether the titanate or zirconate behavior would be dominating.

First let us discuss the Er_2_Ti_2_O_7_ sample and its response to irradiation. Initial characterization showed the pristine sample to be very typical of those synthesized by others, it was highly crystalline with some porosity and an average grain size of approximately 2 microns according to the EBSD images. Analysis of both the synchrotron and neutron diffraction patterns yielded results that were consistent with those reported in the literature with no inherent strain and therefore it can be considered an archetypal pristine example. Irradiation *via* He^2+^ ions resulted in the sample breaking into smaller pieces once it was removed from the mount, this could be due to many reasons that include internal hydrostatic stresses that built up because of the thin nature of the sample, He^2+^ migrating to grain boundaries during irradiation due to insolubility of He^2+^ in pyrochlore, swelling of the irradiated side due to amorphization, volume change due to the formation of helium bubbles or all of the above. Rietveld quantitative phase analysis of the irradiated sample indicated that approximately 30% of the sample had become amorphous which is reasonable given the depth to which it was irradiated and the target dpa and the errors mentioned in the analysis technique. It is also highly likely that the hydrostatic stress field that extended below the predicted depth (Bertarelli, 2014) may have contributed to the amorphization process. Examination *via* EBSD to compare predicted damage depth with degradation in EBSD pattern was not conclusive since the irradiated grains still contained enough coherent areas to produce kikuchi lines that could be indexed *via* the cif generated from neutron diffraction analysis of the pristine sample. This assumption was confirmed *via* TEM, on a sample lifted out from the middle of the predicted damage region. Examination *via* high resolution TEM (Figure 8) indicated that there are regions that are intermixed with disorder and lattice fringes. When a FFT was performed on this region (Figure 8) diffraction spots could be seen in conjunction with a diffuse halo. An image taken at higher magnification (Figure 9) shows these intermixed regions of disorder that are reminiscent of the maze-like patterns in bulk metallic glasses which may lead to the formation of nano crystals resulting from annealing as reported in bulk metallic glasses by Sarac et al. (2016). This result agrees with the neutron diffraction analysis in that there are areas that have become amorphous while others are essentially no different from the starting material. This is therefore a possible explanation as to why no strain or stress was detected in the irradiated sample since any part that might exhibit those properties has become amorphous and any residual stress has been relieved through the fracturing of the sample. It is also of interest that the mosaic pattern produced by the irradiation is very similar to that predicted for the damage of alloys by Simeone et al. (2019). While the material has become amorphous with respect to normal scattering methods there still exists a pattern to the arrangement of the atoms.

The SEM-EBSD results for Nd_2_Zr_2_O_7_ were like those of the Er_2_Ti_2_O_7_ sample in that it was highly crystalline with some porosity. However, there was a larger grain size distribution, most likely because Zr pyrochlores are harder to sinter due to their refractory nature. Prior to irradiation, the synchrotron and neutron diffraction patterns yielded results that were consistent with those reported in the literature with no inherent strain and therefore it can also be considered an archetypal example. In comparison to the Er_2_Ti_2_O_7_ sample upon irradiation the Nd_2_Zr_2_O_7_ sample did not break apart when removed from the sample mount which intuitively suggests that there was a mechanism at play allowing it to accommodate the radiation damage. Rietveld analysis of the neutron diffraction data proved clear evidence of the co-existence of two pyrochlore phases, not a pyrochlore and defect fluorite phase as might be suggested when considering the multiple glancing incidence XRD studies. One of the phases appeared to be very similar to the undamaged material while the other had a lattice parameter that was increased as might be expected in a diffraction pattern exhibiting residual stress. While neutron diffraction data revealed no evidence of strain in either of the phases there is evidence of an increase in disorder of both the cations and anions as shown in Table 2 and Supplementary Figure S6. The method to determine hydrostatic stress as outlined yielded values of hydrostatic residual stress; 1.141 MPa for phase 1 and −0.105 MPa for phase 2, indicating that phase 1 is under tensile stress and that phase 2 is under a very small amount of compressive stress and still fairly like the pristine pyrochlore sample. Considering the studies mentioned previously this is indicative of a ballistic hydrostatic stress field moving deeper into the sample. For comparison it is a well known phenomena that H^+^ migrates deeper into zirconium alloys under the effects of hydrostatic stress (Motta et al., 2019).

Examination *via* EBSD to compare this damaged and undamaged area in Nd_2_Zr_2_O_7_ at first showed very little difference as seen in Figure 5. However, when high resolution EBSD in Figure 6 was performed utilizing both cif files derived from neutron diffraction it was apparent that some grains were composed of high proportions of both phases on a very fine scale. The area highlighted in Figure 5 was prepared for TEM examination *via* FIB to obtain a better understanding of the nature of this region. First the upper area which would be considered to not have any residual stress due to the presence of only phase 2 was examined. This area proved to be highly crystalline with very little disorder as can be seen in Figure 10. In contrast the grain assumed to have a large amount of residual stress due to the presence of both phases exhibited discrete islands of disorder as shown in Figure 13 and Figure 14. FFT of this area resulted in a pattern composed of sharp spots in an area of diffuse scattering with the absence of a halo as seen in the Er_2_Ti_2_O_7_ sample. A higher magnification image reveals that very disordered islands exist in an otherwise highly ordered lattice. There are no regions exhibiting the maze-like pattern as seen in the Er_2_Ti_2_O_7_ sample. It is possible that the highly disordered region would not be able to contribute to the coherent scattering and most likely is imparting stress to the surrounding lattice resulting in reduction in the lattice parameter as damage accumulates. If we consider the events of each ion interaction as a localised heating event there is a parallel with this process and the thermal cycling of thermal barrier coatings and the buildup of residual thermal stresses accumulated during thermal cycling as reported by Guo et al. (2016) for La_2_Zr_2_O_7_. Given that this sample has undergone only a small amount of radiation damage as compared to many of the studies reported in the literature it is conceivable that as more damage occurs the residual stress would continue to build up until the point where the grain fractures due to the pressure front as described earlier. Conversely it could be similar to the process outlined by L M Wang et al. (2000) in their *ex-situ* irradiation study of Ca_2_La_8_(SiO_4_)_6_O_2_. They reported nanostructure formation with a random orientation induced in ceramics by ion beam irradiation at temperatures near the critical amorphization temperature, below the normal crystallization temperature of corresponding amorphous materials. The nanostructure formation is a competition between amorphization and thermally activated recrystallization. In a Ca_2_La_8_(SiO_4_)_6_O_2_, nanocrystals were induced by 1.5 MeV Kr^+^ irradiation at a fluence of 1 × 10^14^ ions/cm^2^ at 673 K, slightly below the critical amorphization temperature. The nanocrystals had the same crystal structure as the original phase as confirmed by electron diffraction. This process has not been viewed in the Nd_2_Zr_2_O_7_ sample however accumulation of amorphous (spherical) regions that may be partly recrystallized would then lead to the scenario where smaller diffraction domains are formed as the grain slowly breaks up due to internal stresses. This process is then analogous to the study of Simeone et al. (2019) where small domains are formed due to grain subdivision with the appearance of a transformation to defect fluorite. A similar process where there is a reduction in *Tc* due to grain size has been reported by Wen et al. (2016) for titanate ceramics. It is therefore possible that with further damage to the zirconate sample the same effect as reported by Simeone et al. would be seen as the damaged grains become progressively divided into smaller coherent scattering domains, thus providing a potential mechanism for the reported transformation of zirconate pyrochlores from pyrochlore to defect fluorite when examined *via* conventional diffraction methods, given x-rays only interact with the upper 2 microns of the sample. Since the titanate based pyrochlore becomes amorphous and does not appear to be able to accommodate more damage and stress, this might be the underlying reason for titanate pyrochlores to transform immediately to the disordered, amorphous state, rather than to the defect fluorite structure when also considered in the context of the study conducted by Wen et al. To extend the argument further this transformation is often simulated through cation substitution in what can be considered type 2 internal strain due to lattice misfit on the atomic length scale. As stated previously our solid solution sample exhibited signs of strain, and thus it is possible that the inherent strain could cause the disruption of the coherent diffraction domains to produce the apparent pyrochlore to defect fluorite transformation. The process could be driven by what is colloquially referred to as compositional strain in solid state chemistry. There are already indications of this mechanism in the literature in various publications that place the defect fluorite transformation boundary at different values of B cation substitution. It is merely a function of how well the samples have been produced to achieve a solid solution to either mitigate or enhance the lattice misfit on the atomic length scale which then results in either their ability to inhibit or enable stress relief.

## Conclusion

We have shown that for both the Er_2_Ti_2_O_7_ and Nd_2_Zr_2_O_7_ pyrochlores strain can’t be detected by peak shape analysis and is most likely regular in nature and that they didn’t undergo a phase transformation to defect fluorite when they are irradiated at levels of approximately 1 dpa *via* He^2+^ ion irradiation at room temperature. The neutron diffraction results indicate an increase in disorder for the irradiated sections of the sample and they still exhibited coherent scattering. There was evidence of hydrostatic stress in the Nd_2_Zr_2_O_7_ sample that was quantified while approximately 30% of the Er_2_Ti_2_O_7_ sample became amorphous. EBSD results confirmed the presence of stress *via* mapping of the two pyrochlore phases throughout certain Nd_2_Zr_2_O_7_ grains to varying degrees. The bulk of both samples remained crystalline to the point of being able to generate kikuchi patterns that could be indexed to the parent phases and so provide orientation information. TEM confirmed the results of the neutron scattering and EBSD while at the same time providing insight into the recovery and radiation response mechanisms of both compositions to irradiation *via* He^2+^ ions. We conclude in a similar fashion as Simeone et al. (2019) that in this study defect fluorite Nd_2_Zr_2_O_7_ did not form and it is indeed possible that it does not form for examples of irradiated zirconate pyrochlores if they behave in a similar fashion. This leads to future experimentational studies: the repetition of our irradiated titanate and zirconate samples to examine the grains that appear to have the highest regions of stress *via* nano Raman spectroscopy to determine if the domains do indeed break down and in-depth stress experiments to confirm if the bulk of the stress is similar in nature to the examples cited. Then subsequently if those sub domains still produce a pattern consistent with pyrochlore as seen by Simeone et al. (2019) with nano-grains of La_2_Zr_2_O_7_ as this will provide additional insights about the here proposed radiation response mechanism for zirconate and titanate pyrochlores. Future work will also focus on the synthesis of a range of pyrochlore solid solutions with mixed B cations to perform similar irradiation experiments coupled with grain size effects.

## Data Availability

The original contributions presented in the study are included in the article/Supplementary Material, further inquiries can be directed to the corresponding authors.
