# Supplementary material for: Insight Into Disorder, Stress and Strain of Radiation Damaged Pyrochlores: A Possible Mechanism for the Appearance of Defect Fluorite
Source: Front Chem. 2021 Nov 8;9:706736. doi: 10.3389/fchem.2021.706736 (PMC8630592; doi:10.3389/fchem.2021.706736)
Supplement: Supplementary file 1 [file DataSheet1.zip › Table 1.DOCX]

Table S1: Density determination of all zirconate and titanate pellets fabricated for the irradiation studies. Densities were determined geometrically for all pellets as well as via Archimedes method for selected samples.

| Sample | Chemical composition | Theoretical density (%) via archimedes | Theoretical density (%) determined geometrically |
| --- | --- | --- | --- |
| RE-303-1 | Nd2Zr2O7 | 97.7 | 83.56 |
| RE-303-2 | Nd2Zr2O7 |  | 83.85 |
| RE-303-3 | Nd2Zr2O7 |  | 83.76 |
| RE-303-4 | Nd2Zr2O7 |  | 84.17 |
| RE-303-5 | Nd2Zr2O7 |  | 83.80 |
| RE-303-6 | Nd2Zr2O7 |  | 84.01 |
| RE-303-7 | Nd2Zr2O7 |  | 84.34 |
| RE-303-8 | Nd2Zr2O7 |  | 83.86 |
| RE-311-1 | Er2Ti2O7 | 92.7 | 88.00 |
| RE-311-2 | Er2Ti2O7 |  | 88.96 |
| RE-311-3 | Er2Ti2O7 |  | 89.37 |
| RE-311-4 | Er2Ti2O7 |  | 89.14 |
| RE-311-5 | Er2Ti2O7 |  | 87.86 |
| RE-311-6 | Er2Ti2O7 |  | 88.92 |
| RE-311-7 | Er2Ti2O7 |  | 88.08 |
| RE-311-8 | Er2Ti2O7 |  | 88.43 |
