# Supplementary material for: Insight Into Disorder, Stress and Strain of Radiation Damaged Pyrochlores: A Possible Mechanism for the Appearance of Defect Fluorite
Source: Front Chem. 2021 Nov 8;9:706736. doi: 10.3389/fchem.2021.706736 (PMC8630592; doi:10.3389/fchem.2021.706736)
Supplement: Supplementary file 1 [file DataSheet1.zip › Table 3.DOCX]

data_VESTA_phase_1

_audit_creation_method 'generated by CrystalMaker X for macOS'

_cell_length_a 10.647150(1)

_cell_length_b 10.647150(1)

_cell_length_c 10.647150(1)

_cell_angle_alpha 90.000000(0)

_cell_angle_beta 90.000000(0)

_cell_angle_gamma 90.000000(0)

_symmetry_space_group_name_H-M 'F d -3 m'

loop_

_symmetry_equiv_pos_as_xyz

'+x,+y,+z'

'+z,+x,+y'

'+y,+z,+x'

'-x,1/4+y,1/4+z'

'-z,1/4+x,1/4+y'

'-y,1/4+z,1/4+x'

'1/4+x,-y,1/4+z'

'1/4+z,-x,1/4+y'

'1/4+y,-z,1/4+x'

'1/4-x,3/4-y,1/2+z'

'1/4-z,3/4-x,1/2+y'

'1/4-y,3/4-z,1/2+x'

'+y,+x,+z'

'+x,+z,+y'

'+z,+y,+x'

'1/4+y,-x,1/4+z'

'1/4+x,-z,1/4+y'

'1/4+z,-y,1/4+x'

'-y,1/4+x,1/4+z'

'-x,1/4+z,1/4+y'

'-z,1/4+y,1/4+x'

'3/4-y,1/4-x,1/2+z'

'3/4-x,1/4-z,1/2+y'

'3/4-z,1/4-y,1/2+x'

'-x,-y,-z'

'-z,-x,-y'

'-y,-z,-x'

'+x,3/4-y,3/4-z'

'+z,3/4-x,3/4-y'

'+y,3/4-z,3/4-x'

'3/4-x,+y,3/4-z'

'3/4-z,+x,3/4-y'

'3/4-y,+z,3/4-x'

'3/4+x,1/4+y,1/2-z'

'3/4+z,1/4+x,1/2-y'

'3/4+y,1/4+z,1/2-x'

'-y,-x,-z'

'-x,-z,-y'

'-z,-y,-x'

'3/4-y,+x,3/4-z'

'3/4-x,+z,3/4-y'

'3/4-z,+y,3/4-x'

'+y,3/4-x,3/4-z'

'+x,3/4-z,3/4-y'

'+z,3/4-y,3/4-x'

'1/4+y,3/4+x,1/2-z'

'1/4+x,3/4+z,1/2-y'

'1/4+z,3/4+y,1/2-x'

'+x,1/2+y,1/2+z'

'1/2+x,1/2+y,+z'

'1/2+x,+y,1/2+z'

'+z,1/2+x,1/2+y'

'1/2+z,1/2+x,+y'

'1/2+z,+x,1/2+y'

'+y,1/2+z,1/2+x'

'1/2+y,1/2+z,+x'

'1/2+y,+z,1/2+x'

'-x,3/4+y,3/4+z'

'1/2-x,3/4+y,1/4+z'

'1/2-x,1/4+y,3/4+z'

'-z,3/4+x,3/4+y'

'1/2-z,3/4+x,1/4+y'

'1/2-z,1/4+x,3/4+y'

'-y,3/4+z,3/4+x'

'1/2-y,3/4+z,1/4+x'

'1/2-y,1/4+z,3/4+x'

'1/4+x,1/2-y,3/4+z'

'3/4+x,1/2-y,1/4+z'

'3/4+x,-y,3/4+z'

'1/4+z,1/2-x,3/4+y'

'3/4+z,1/2-x,1/4+y'

'3/4+z,-x,3/4+y'

'1/4+y,1/2-z,3/4+x'

'3/4+y,1/2-z,1/4+x'

'3/4+y,-z,3/4+x'

'1/4-x,1/4-y,+z'

'3/4-x,1/4-y,1/2+z'

'3/4-x,3/4-y,+z'

'1/4-z,1/4-x,+y'

'3/4-z,1/4-x,1/2+y'

'3/4-z,3/4-x,+y'

'1/4-y,1/4-z,+x'

'3/4-y,1/4-z,1/2+x'

'3/4-y,3/4-z,+x'

'+y,1/2+x,1/2+z'

'1/2+y,1/2+x,+z'

'1/2+y,+x,1/2+z'

'+x,1/2+z,1/2+y'

'1/2+x,1/2+z,+y'

'1/2+x,+z,1/2+y'

'+z,1/2+y,1/2+x'

'1/2+z,1/2+y,+x'

'1/2+z,+y,1/2+x'

'1/4+y,1/2-x,3/4+z'

'3/4+y,1/2-x,1/4+z'

'3/4+y,-x,3/4+z'

'1/4+x,1/2-z,3/4+y'

'3/4+x,1/2-z,1/4+y'

'3/4+x,-z,3/4+y'

'1/4+z,1/2-y,3/4+x'

'3/4+z,1/2-y,1/4+x'

'3/4+z,-y,3/4+x'

'-y,3/4+x,3/4+z'

'1/2-y,3/4+x,1/4+z'

'1/2-y,1/4+x,3/4+z'

'-x,3/4+z,3/4+y'

'1/2-x,3/4+z,1/4+y'

'1/2-x,1/4+z,3/4+y'

'-z,3/4+y,3/4+x'

'1/2-z,3/4+y,1/4+x'

'1/2-z,1/4+y,3/4+x'

'3/4-y,3/4-x,+z'

'1/4-y,3/4-x,1/2+z'

'1/4-y,1/4-x,+z'

'3/4-x,3/4-z,+y'

'1/4-x,3/4-z,1/2+y'

'1/4-x,1/4-z,+y'

'3/4-z,3/4-y,+x'

'1/4-z,3/4-y,1/2+x'

'1/4-z,1/4-y,+x'

'-x,1/2-y,1/2-z'

'1/2-x,1/2-y,-z'

'1/2-x,-y,1/2-z'

'-z,1/2-x,1/2-y'

'1/2-z,1/2-x,-y'

'1/2-z,-x,1/2-y'

'-y,1/2-z,1/2-x'

'1/2-y,1/2-z,-x'

'1/2-y,-z,1/2-x'

'+x,1/4-y,1/4-z'

'1/2+x,1/4-y,3/4-z'

'1/2+x,3/4-y,1/4-z'

'+z,1/4-x,1/4-y'

'1/2+z,1/4-x,3/4-y'

'1/2+z,3/4-x,1/4-y'

'+y,1/4-z,1/4-x'

'1/2+y,1/4-z,3/4-x'

'1/2+y,3/4-z,1/4-x'

'3/4-x,1/2+y,1/4-z'

'1/4-x,1/2+y,3/4-z'

'1/4-x,+y,1/4-z'

'3/4-z,1/2+x,1/4-y'

'1/4-z,1/2+x,3/4-y'

'1/4-z,+x,1/4-y'

'3/4-y,1/2+z,1/4-x'

'1/4-y,1/2+z,3/4-x'

'1/4-y,+z,1/4-x'

'3/4+x,3/4+y,-z'

'1/4+x,3/4+y,1/2-z'

'1/4+x,1/4+y,-z'

'3/4+z,3/4+x,-y'

'1/4+z,3/4+x,1/2-y'

'1/4+z,1/4+x,-y'

'3/4+y,3/4+z,-x'

'1/4+y,3/4+z,1/2-x'

'1/4+y,1/4+z,-x'

'-y,1/2-x,1/2-z'

'1/2-y,1/2-x,-z'

'1/2-y,-x,1/2-z'

'-x,1/2-z,1/2-y'

'1/2-x,1/2-z,-y'

'1/2-x,-z,1/2-y'

'-z,1/2-y,1/2-x'

'1/2-z,1/2-y,-x'

'1/2-z,-y,1/2-x'

'3/4-y,1/2+x,1/4-z'

'1/4-y,1/2+x,3/4-z'

'1/4-y,+x,1/4-z'

'3/4-x,1/2+z,1/4-y'

'1/4-x,1/2+z,3/4-y'

'1/4-x,+z,1/4-y'

'3/4-z,1/2+y,1/4-x'

'1/4-z,1/2+y,3/4-x'

'1/4-z,+y,1/4-x'

'+y,1/4-x,1/4-z'

'1/2+y,1/4-x,3/4-z'

'1/2+y,3/4-x,1/4-z'

'+x,1/4-z,1/4-y'

'1/2+x,1/4-z,3/4-y'

'1/2+x,3/4-z,1/4-y'

'+z,1/4-y,1/4-x'

'1/2+z,1/4-y,3/4-x'

'1/2+z,3/4-y,1/4-x'

'1/4+y,1/4+x,-z'

'3/4+y,1/4+x,1/2-z'

'3/4+y,3/4+x,-z'

'1/4+x,1/4+z,-y'

'3/4+x,1/4+z,1/2-y'

'3/4+x,3/4+z,-y'

'1/4+z,1/4+y,-x'

'3/4+z,1/4+y,1/2-x'

'3/4+z,3/4+y,-x'

loop_

_atom_site_label

_atom_site_type_symbol

_atom_site_occupancy

_atom_site_fract_x

_atom_site_fract_y

_atom_site_fract_z

_atom_site_U_iso_or_equiv

Nd1 Nd 1.0 0.50000000(0) 0.50000000(0) 0.50000000(0) 0.00830

O1 O 1.0 0.33670000(40000) 0.12500000(0) 0.12500000(0) 0.02140

O2 O 1.0 0.37500000(0) 0.37500000(0) 0.37500000(0) 0.00100

Zr1 Zr 1.0 0.00000000(0) 0.00000000(0) 0.00000000(0) 0.01260

loop_

_atom_site_aniso_label

_atom_site_aniso_U_11

_atom_site_aniso_U_22

_atom_site_aniso_U_33

_atom_site_aniso_U_23

_atom_site_aniso_U_13

_atom_site_aniso_U_12

Nd1 0.00830 0.00830 0.00830 0.00110 0.00110 0.00110

O1 0.02140 0.02140 0.02140 -0.00030 0.00000 0.00000

O2 0.00100 0.00100 0.00100 0.00000 0.00000 0.00000

Zr1 0.01260 0.01260 0.01260 0.00770 0.00770 0.00770
