# Supplementary material for: Insight Into Disorder, Stress and Strain of Radiation Damaged Pyrochlores: A Possible Mechanism for the Appearance of Defect Fluorite
Source: Front Chem. 2021 Nov 8;9:706736. doi: 10.3389/fchem.2021.706736 (PMC8630592; doi:10.3389/fchem.2021.706736)
Supplement: Supplementary file 1 [file DataSheet1.zip › Supplementary Figures S1 - S8.docx]

# *Supplementary Material*


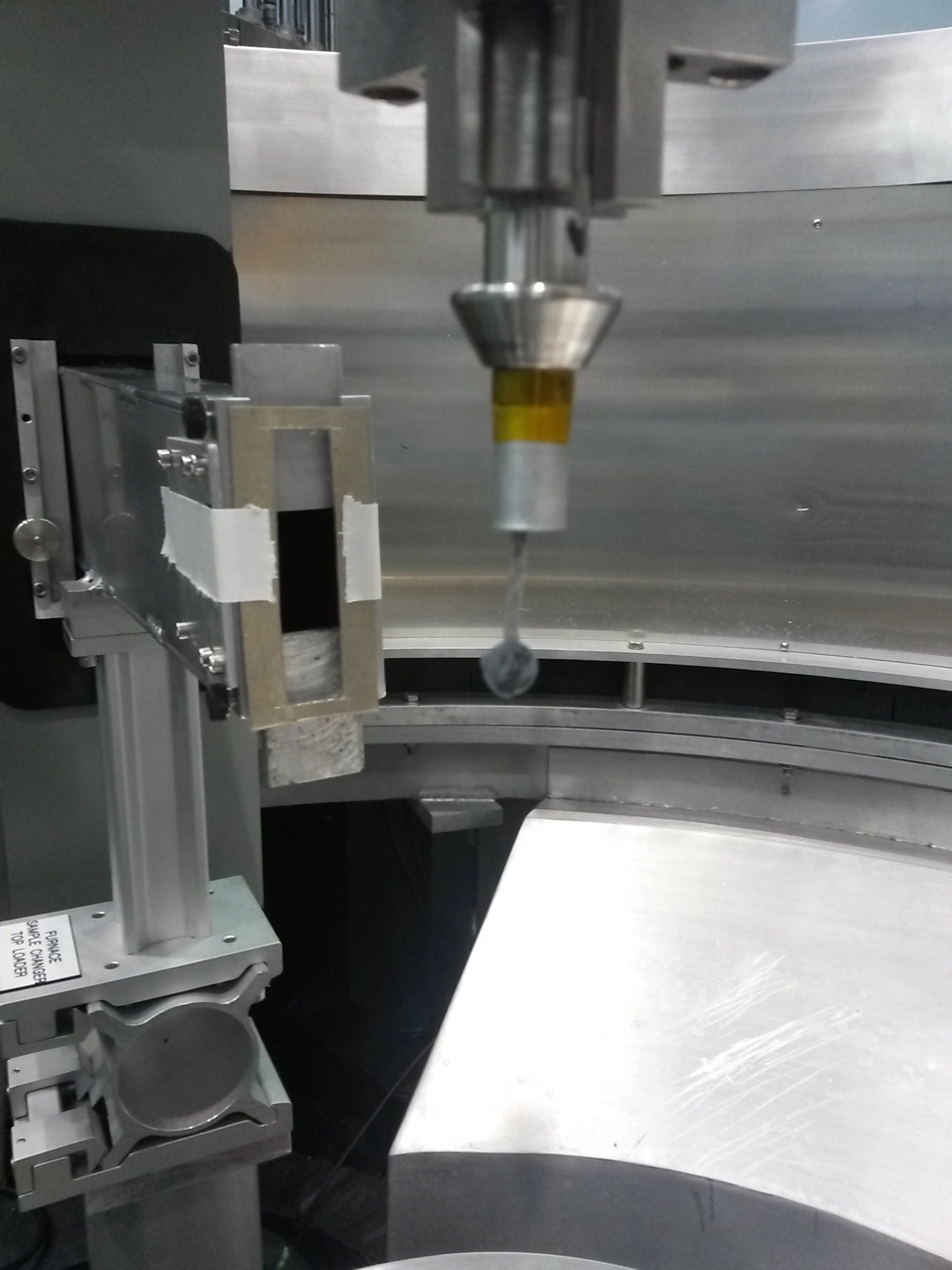


Supplementary Figure S1: Nd_2_Zr_2_O_7_ sample mounted for neutron diffraction on the Echidna high resolution diffractometer.


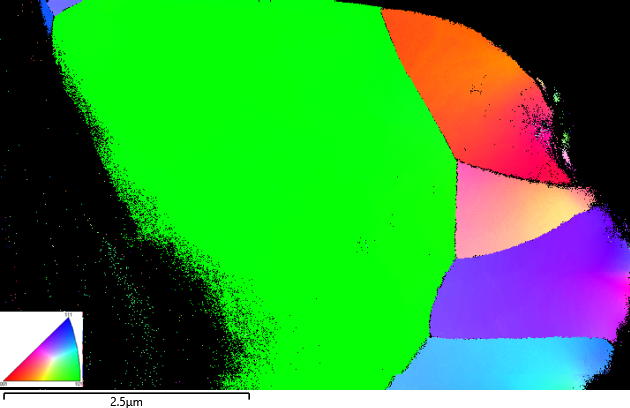


Supplementary Figure S2: High resolution EBSD of Nd_2_Zr_2_O_7_ lamella. Loss of edge signal in crystal shaded green is due to the crystal curving off at the edge.


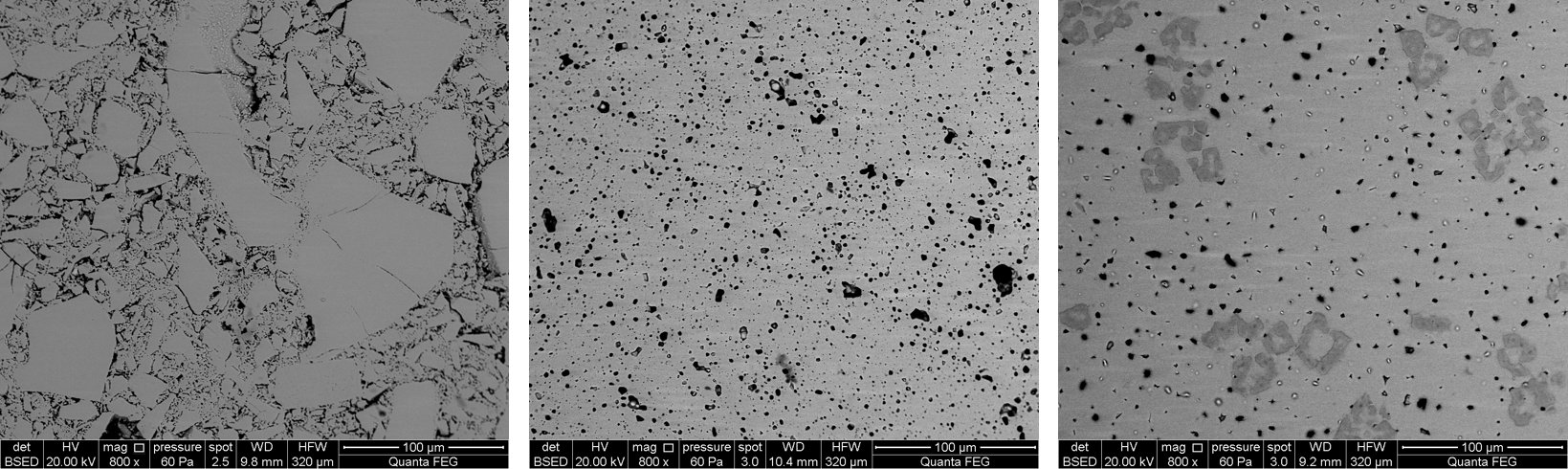


Supplementary figure S3: SEM images of the Nd_2_Zr_2_O_7_ (left), Er_2_Ti_2_O_7_ (middle), and (NdEr)_2_(ZrTi)_2_O_7_ (right) sintered pellets.


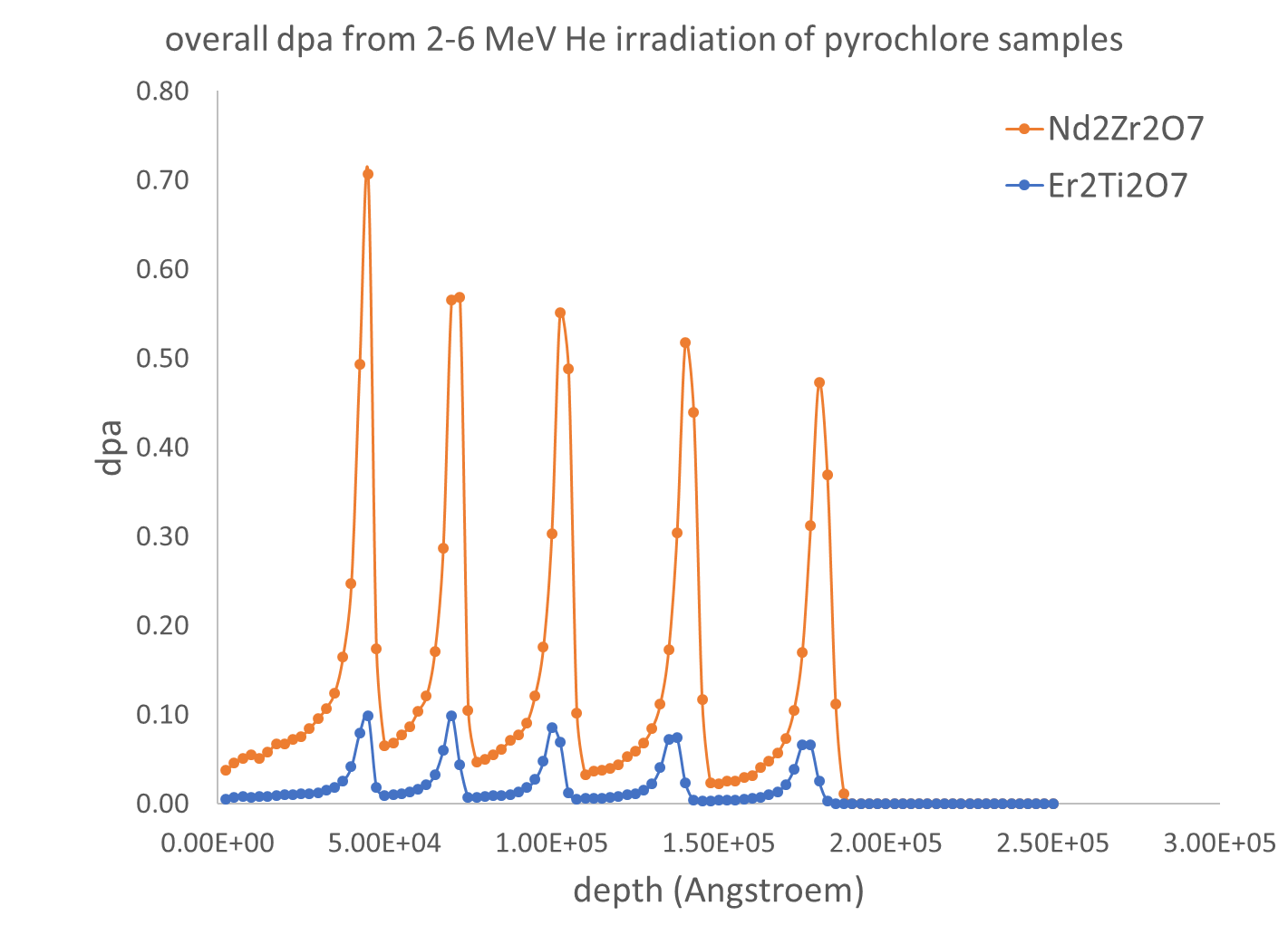


Supplementary figure S4: Sum of TRIM calculated displacements per atom for a Nd_2_Zr_2_O_7_ pyrochlore (orange) and an Er_2_Ti_2_O_7_ (blue) sample for subsequent He ion irradiation with 2, 3, 4 ,5 and 6 MeV with a fluence of 1x10^16^ ions/cm^2^.


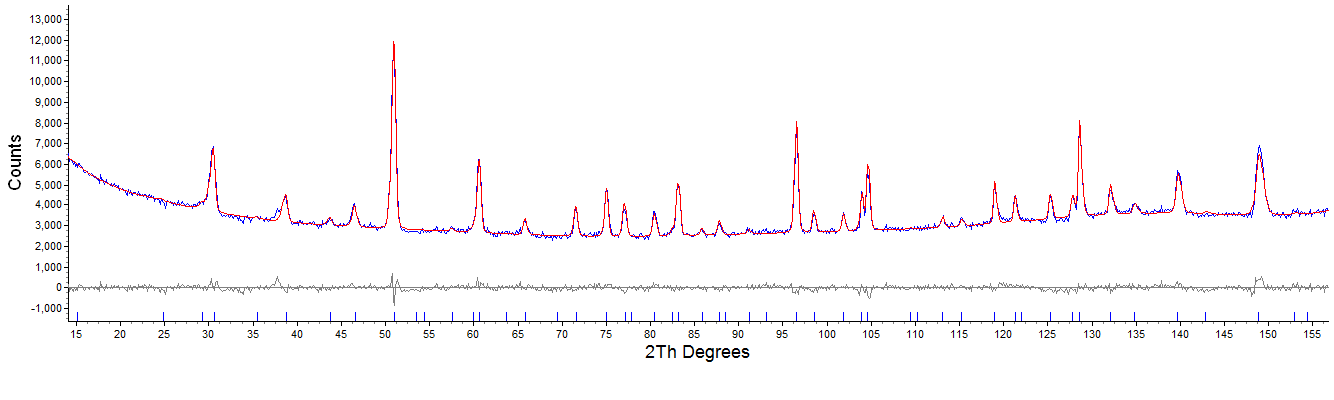


Supplementary figure S5: Rietveld refinement of undamaged Nd_2_Zr_2_O_7_ showing quality of fit to a single phase.


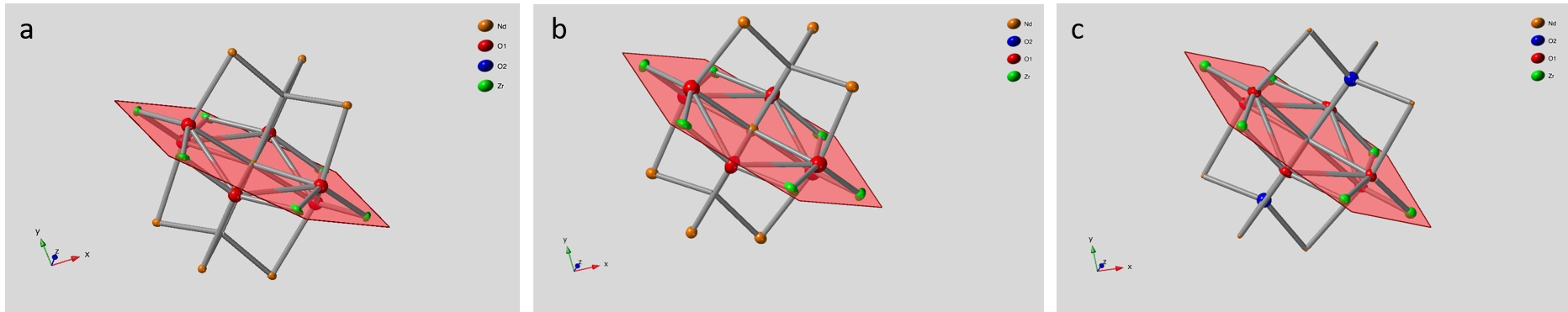


Supplementary figure S6: (a) Unirradiated Nd_2_Zr_2_O_7_ sample with anisotropic displacement parameter ellipsoids plotted and the 111 plane highlighted, (b) irradiated Nd_2_Zr_2_O_7_ sample phase 1 with anisotropic displacement parameter ellipsoids plotted and the 111 plane highlighted, and (c) irradiated Nd_2_Zr_2_O_7_ sample phase 2 with anisotropic displacement parameter ellipsoids plotted and the 111 plane highlighted.


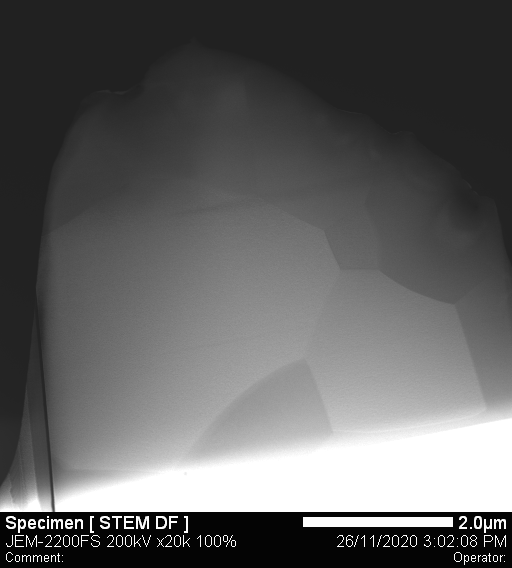


Supplementary figure S7: Corresponding area of figure 9 imaged in STEM mode with an annular dark field detector.


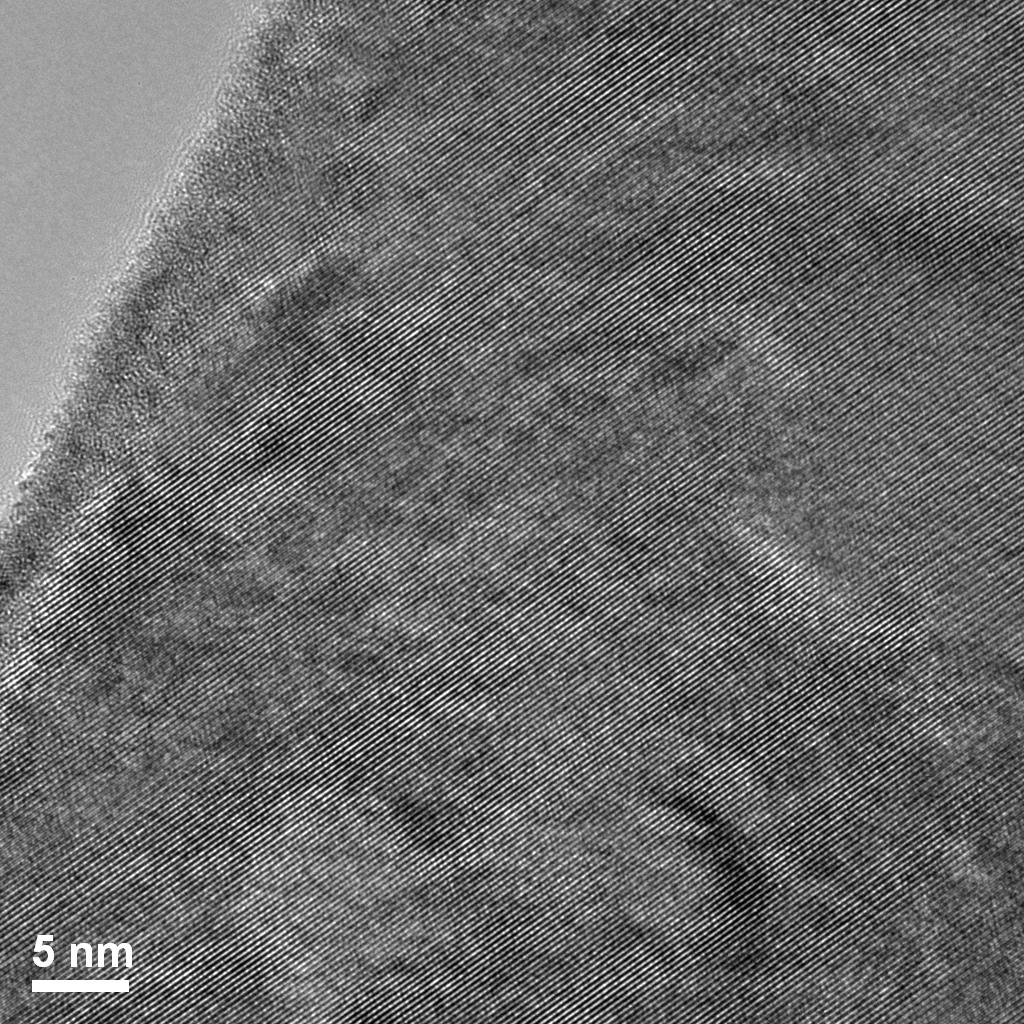


Supplementary figure S8: Edge of lamella for the Nd2Zr2O7 sample showing a thin amorphous region of approximate dimensions as predicted by Mayer et al. (Mayer et al., 2007)
